# Photo-click hydrogels for 3D in situ differentiation of pancreatic progenitors from induced pluripotent stem cells

**DOI:** 10.1186/s13287-023-03457-7

**Published:** 2023-08-30

**Authors:** Matthew R. Arkenberg, Yoshitomo Ueda, Eri Hashino, Chien-Chi Lin

**Affiliations:** 1 Weldon School of Biomedical Engineering, Purdue University, West Lafayette, IN 47907 USA; 2 Department of Otolaryngology-Head and Neck Surgery, Indiana University School of Medicine, Indianapolis, IN 46202 USA; 3 Stark Neurosciences Research Institute, Indiana University School of Medicine, Indianapolis, IN 46202 USA; 4 Department of Biomedical Engineering, Indiana University-Purdue University Indianapolis, 723 W. Michigan St. SL220K, Indianapolis, IN 46202 USA; 5 Indiana University Simon Comprehensive Cancer Center, Indianapolis, IN 46202 USA

**Keywords:** Induced pluripotent stem cells, Pancreatic progenitor, Hydrogels, Photo-click chemistry, Single-cell RNA-sequencing, Wnt signaling

## Abstract

**Background:**

Induced pluripotent stem cells (iPSC) can be differentiated to cells in all three germ layers, as well as cells in the extraembryonic tissues. Efforts in iPSC differentiation into pancreatic progenitors in vitro have largely been focused on optimizing soluble growth cues in conventional two-dimensional (2D) culture, whereas the impact of three-dimensional (3D) matrix properties on the morphogenesis of iPSC remains elusive.

**Methods:**

In this work, we employ gelatin-based thiol-norbornene photo-click hydrogels for in situ 3D differentiation of human iPSCs into pancreatic progenitors (PP). Molecular analysis and single-cell RNA-sequencing were utilized to elucidate on the distinct identities of subpopulations within the 2D and 3D differentiated cells.

**Results:**

We found that, while established soluble cues led to predominately PP cells in 2D culture, differentiation of iPSCs using the same soluble factors led to prominent branching morphogenesis, ductal network formation, and generation of diverse endoderm populations. Through single-cell RNA-sequencing, we found that 3D differentiation resulted in enrichments of pan-endodermal cells and ductal cells. We further noted the emergence of a group of extraembryonic cells in 3D, which was absent in 2D differentiation. The unexpected emergence of extraembryonic cells in 3D was found to be associated with enrichment of Wnt and BMP signaling pathways, which may have contributed to the emergence of diverse cell populations. The expressions of PP signature genes *PDX1* and *NKX6.1* were restored through inhibition of Wnt signaling at the beginning of the posterior foregut stage.

**Conclusions:**

To our knowledge, this work established the first 3D hydrogel system for in situ differentiation of human iPSCs into PPs.

**Supplementary Information:**

The online version contains supplementary material available at 10.1186/s13287-023-03457-7.

## Background

Human-induced pluripotent stem cells (iPSCs) are capable of being differentiated into cells in all three germ layers—ectoderm, mesoderm, and endoderm [1–3]. Recent studies have also shown that iPSCs can be primed to undergo trophoblastic differentiation [4]. Efficient generation of pancreatic cell lineages requires that iPSCs be first differentiated into definitive endoderm (DE) cells, which is facilitated by using Activin A, a member of the transforming growth factor β (TGF-β) superfamily [5]. Successful DE differentiation is characterized by the expression of SOX17, an endoderm-associated transcription factor. Subsequent differentiation steps include the induction of primitive gut tube (PGT), posterior foregut (PF), and finally pancreatic progenitors (PP) cells. Cells expressing pancreatic and duodenal homeobox 1 (PDX1) and NKX6 homeobox 1 (NKX6.1) are considered multipotent PP cells. Further differentiation of multipotent PP cells leads to endocrine pancreatic islets composed primarily of α-, β-, γ-cells, as well as exocrine cells (i.e., pancreatic duct and acinar cells) that organized into a branching, polarized, and lumenized structure [6]. Attempts to recapitulate these embryonic developmental stages are primarily focused on optimizing the contents of soluble cues supplied in conventional two-dimensional (2D) cell culture vessels, which present unnaturally stiff and non-compliant environment [6]. However, the fate of embryonic cell development is also guided by the interactions between the developing cells with the surrounding matrices, which present varying stiffness and matrix compositions throughout the developmental stages. As such, engineered three-dimensional (3D) matrices may provide a more biomimetic context where cells can self-organize and interact with their local microenvironment [7]. 3D cell cultures permit not only multi-axial cell–cell interactions, but also cell-extracellular matrix (ECM) interactions that are proven to influence the differentiation of PSCs into other cell types [8–10]. To this end, engineered hydrogels are widely used as synthetic and biomimetic matrices for 3D cell culture, including embryonic stem cells (ESCs) and iPSCs. To the best of our knowledge, engineered hydrogels have not been used to study the effect of matrix properties on iPSC-derived PP differentiation.

Current protocols for PP differentiation have primarily focused on generating PDX1/NKX6.1 positive cells, followed by maturation of PP cells into endocrine cells by transplanting the immature cells in vivo [11–13]. Maturation of endocrine cells can also be achieved by seeding iPSC-derived PP cells on or within hydrogels consisting of type 1 collagen, decellularized ECM (dECM), or tumor-derived Matrigel [14]. For example, Hogrebe and colleagues showed that increasing collagen substrate thickness led to softer matrix that encouraged endocrine induction of PP cells [15]. In another example, Breunig and coworkers demonstrated that PP cells encapsulated in Matrigel facilitated their differentiation into pancreatic duct-like organoids [16]. With respect to pluripotent stem cells to PP cell differentiation in 3D, Richardson and colleagues studied pancreatic differentiation of ESCs using barium-crosslinked alginate hydrogels with different crosslinking density [10]. Notably, stiffer hydrogels enhanced endoderm differentiation but suppressed pancreatic differentiation, whereas softer hydrogels yielded cells with high *PDX1* expression. Interestingly, *PTF1A*, another pancreatic differentiation marker, was significantly upregulated in stiffer alginate gels. Unfortunately, *NKX6.1*, an important pancreatic progenitor marker, was not quantified in this study. Further, this study did not examine the effect of cell-ECM interactions on PP cell differentiation.

We have previously shown that photo-click thiol-norbornene hydrogels supported iPSC culture and DE differentiation [17, 18]. In particular, we developed synthetic and dynamically tunable hydrogels from poly(ethylene glycol)-norbornene (PEGNB) and protease-labile peptide crosslinker to support xeno-free growth and DE differentiation of iPSCs [17]. We further integrated orthogonal thiol-norbornene and norbornene–tetrazine click chemistries for constructing biologically derived gelatin-norbornene (GelNB) hydrogels to support tri-germ layer differentiation of iPSCs [18]. In particular, we showed that naturally derived basement membrane matrix (Geltrex) was incapable of supporting in situ proliferation and differentiation of iPSCs. Using orthogonally crosslinked GelNB hydrogels with independently tunable bioactive motifs and matrix stiffness, we demonstrated that viability of iPSC in 3D hydrogels negatively correlated with hydrogel stiffness. We also observed that GelNB hydrogels with shear moduli (G′) of 1.5 kPa were suited for in situ 3D differentiation of iPSCs into neuroectoderm, mesoderm, and definitive endoderm.

Prior results have shown that hydrogels/substrates with higher stiffness supported definitive endoderm differentiation but significantly hampered subsequent endocrine cell maturation [10, 15]. However, in our prior work, we showed that chemically crosslinked hydrogels with higher stiffness (G′ > 2 kPa) were not ideal for in situ proliferation and differentiation of iPSCs [18]. Given that softer GelNB hydrogels were supportive of DE differentiation, we sought to explore this photo-click hydrogel system for in situ proliferation and differentiation of iPSCs into PP cells. Under the same gelatin content, we investigated the effect of matrix stiffness on expression of foregut progenitor and pancreatic progenitor markers. Through immunostaining and single-cell transcriptomic analyses, we characterized morphology of the multi-cellular structures and identified populations of differentiated cells following the 4-stage PP differentiation protocol. In 3D GelNB hydrogels, we further discovered a notable enrichment of extraembryonic cell populations, which were not observed in conventional 2D differentiated cells. The emergence of extraembryonic cells was accompanied by elevated Wnt and BMP signaling. Finally, we restored PP cell differentiation in 3D by inhibiting Wnt and BMP pathways.

## Materials and methods

### Materials

Four-arm PEGSH (10 kDa) was purchased from JenKem Technology and Laysan Bio, respectively. Type A Gelatin was purchased from Amresco. Carbic anhydride and 1-(3-dimethylaminopropyl)-3-ethylcarbodiimide hydrochloride (EDC) were purchased from Acros. N-hydroxysuccinimide was purchased from TCI. Photoinitiator lithium aryl phosphinate (LAP) was obtained from Sigma-Aldrich. All other chemicals were purchased from Fisher Scientific unless noted otherwise.

### Mechanical characterization of GelNB hydrogels

Macromer GelNB was synthesized as described previously [19, 20]. Photo-click hydrogels were fabricated by radical initiated thiol-norbornene photopolymerization. All gelatin-based hydrogels were crosslinked by 5 wt% GelNB and PEGSH (0.6 wt% for soft and 1.0 wt% for stiff GelNB gels). All hydrogels were polymerized in the presence of LAP (2 mM) and 2-min longwave UV-light (365 nm, 5 mW/cm^2^). After crosslinking, all gels were placed in sterile Dulbecco’s phosphate buffered saline (DPBS) at 37 °C overnight to achieve equilibrium swelling. Oscillatory rheometry was performed on a Bohlin CV100 digital rheometer. Strain sweep mode was utilized to obtain elastic and loss moduli (G′ and G′′).

### Maintenance and encapsulation of human iPSCs

Cellartis hiPSC12 cell line (ChiPSC12, Takara) was cultured on vitronectin (VTN)-coated tissue plates in Essential 8^TM^ (E8, Gibco) medium. VTN coating was conducted per manufacturer’s protocol without modification. The media was supplemented with 10 µM ROCK inhibitor, Y-27632 (E8Y, Adipogen) for the first 24 h following thawing or passaging to prevent anoikis. Media were refreshed daily, and cells were passaged every 3–4 days with 5 min treatment of TrypLE^TM^ Select dissociation reagent (Gibco).

Cells were encapsulated in the hydrogels with formulations described above. All precursor components were mixed prior to adding the iPSCs to a final cell density of 2 × 10^6^ cells/mL. hiPSC-containing precursor solutions were mixed gently and pipetted into a cylindrical syringe mold and placed under 365 nm light (5 mW/cm^2^) for 2 min. Following gelation, the samples were transferred to a 24-well plate containing E8Y media. The E8Y media was refreshed on day 2 post-encapsulation,

### DE and PP differentiation in 2D and 3D thiol-ene crosslinked hydrogels

**Fig. 1:**
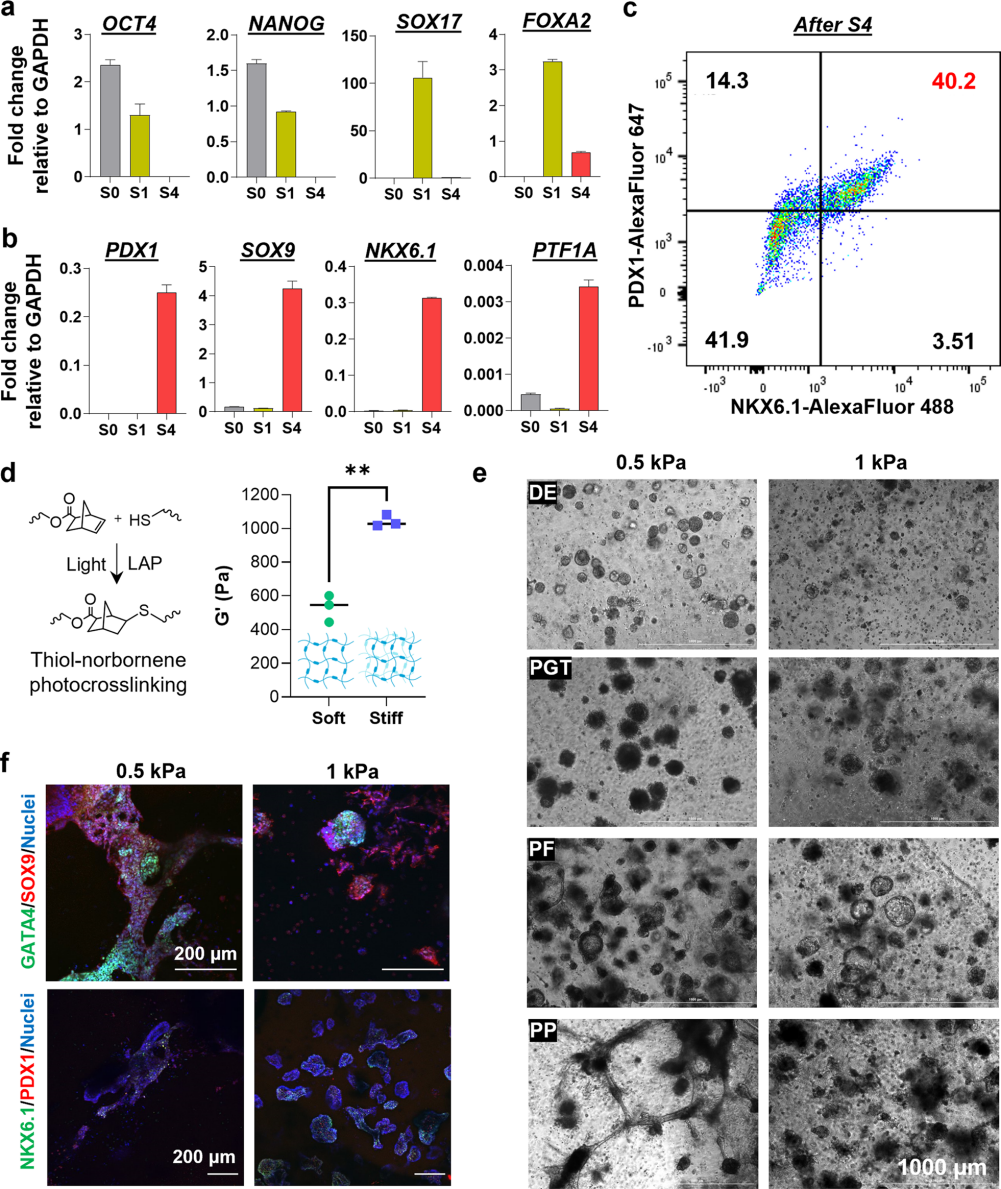
Pancreatic differentiation of iPSCs in 3D led to prominent tubular network. **a** Expression of pluripotency genes *OCT4*/*NANOG* and DE signature genes *SOX17*/*FOXA2* during S0, S1, and S4 of PP differentiation in 2D culture. **b** Expression of PP signature genes *PDX1*, *SOX9*, *NKX6.1*, and *PTF1A* in S0, S1, and S4 of PP differentiation in 2D culture. **c** Flow cytometry results of 2D-differentiated S4 cells stained with PDX1 and NKX6.1. **d** Schematic of thiol-norbornene photo-click reaction to form covalent thioether crosslink. **e** Shear moduli of GelNB hydrogels for 3D PP differentiation. 5 wt% GelNB was crosslinked by either 0.6 wt% or 1.0 wt% PEG4SH to create hydrogels with 0.5 kPa or 1 kPa shear moduli. A two-tailed t-test was utilized for statistical analysis (** represents *p*-value < 0.001). **f** Effect of GelNB hydrogel stiffness on the morphology of 3D encapsulated cells after DE (S1), PGT (S2), PF (S3), and PP (S4) differentiation. **g** Effect of 3D GelNB hydrogel stiffness on SOX9, GATA4, NKX6.1, and PDX1 expression. At least three representative images were taken (10 slices in each z-stack with a slice height of 10 µm)

All differentiation studies were conducted using a commercial pancreatic progenitor differentiation kit (STEMCELL Technologies, Fig. 1A). 2D differentiation studies were conducted following manufacturer’s protocol without modification. For 3D differentiation, hiPSCs were encapsulated as single cells and cultured in hydrogels until day 4, when pancreatic differentiation was initiated. DE and PP lineage commitment was assessed 2 days and 14 days after starting differentiation, respectively. Cell morphology was assessed by taking brightfield images at the end of each stage of differentiation, i.e., day 4, 6, 9, 12, and 17 for iPSC, DE, PGT, PF, and PP stages, respectively. For Wnt and BMP inhibition studies, either XAV939 (2 μM, Selleck Chemicals) or LDN193189 (200 nM, Selleck Chemicals) or both were added to the media at the start of the PGT stage or PF stage as specified. For the vehicle control, equivalent volume of DMSO was added. Inhibitors and vehicle control media were refreshed daily. Live/dead staining and confocal imaging was conducted at the iPSC, DE, and PP stages with 1 h incubation followed by three 5-min washes with DPBS. Imaging was conducted on a confocal microscope (Olympus Fluoview FV100 laser scanning microscope) with at least three regions of interest per sample and at least 100 μm z-stack thickness.

### Immunofluorescence staining and imaging

At specified time points, cell-laden hydrogels were washed once with DPBS for 5 min and fixed with paraformaldehyde (PFA, 4%) for 45 min at room temperature. The fixed hydrogels were then washed once with DPBS and twice with blocking buffer composed of 1% bovine serum albumin (BSA) and 0.3% Triton X-100 for 5 min each at room temperature. The gels were treated with blocking buffer for 1 h at room temperature followed by incubation overnight at 4 °C in blocking buffer containing primary antibodies (Additional file 1: Table S1). After three 45-min washes with blocking buffer, the samples were incubated overnight with the corresponding secondary antibodies (Additional file 1: Table S1). After three additional 30-min washes with DPBS, the samples were counterstained with DAPI nuclear stain for 1 h, followed by three 10-min washes with DPBS. Imaging was conducted using a confocal microscope (Olympus Fluoview FV100 laser scanning microscope) with at least three regions of interest per sample and at least 100 μm z-stack thickness. Quantification of corrected total fluorescence was conducted using ImageJ software. In brief, the corrected total fluorescence (CTF) for each channel was determined by subtracting the area of a selected region multiplied by the mean fluorescence of the background readings from the integrated density. The CTF values for NKX6.1 and PDX1 were normalized to the nuclear CTF value. Three images per condition were assessed with three selected regions per image.

### Flow cytometry of pancreatic progenitor cells differentiated in 2D culture

After 2D PP differentiation, cells were dissociated using TrypLE Select dissociation reagent, washed once with DPBS with centrifugation (1000 rpm for 3 min), and incubated with 1% PFA for 15 min. Following three washes with DPBS and centrifugation, the samples were blocked with blocking/permeabilization buffer for 1 h. Primary antibodies (Additional file 1: Table S1) were added to the samples and incubated at 4 °C overnight. Three washes with blocking/permeabilization buffer and centrifugation were conducted followed by addition of secondary antibodies for one hour incubation. Stained samples were washed three additional times with DPBS followed by filtration with 40 μm cell strainer and flow cytometry on an BD LSR II flow cytometer. Analysis was conducted using FlowJo™ software.

### RNA extraction, reverse transcription (RT), and quantitative PCR (qPCR) analysis

At specified time points, three hydrogels were pooled and incubated with Type 1 collagenase (50 U/mL) for 1 h to liberate the cells. After one wash with DPBS, RNA was extracted with a NucleoSpin RNA II kit (Clontech) per manufacturer’s protocol. A NanoDrop 2000 spectrophotometer (Thermo Scientific) was utilized to quantify RNA quality and concentration. RT-PCR was conducted using a PrimeScript RT reagent kit (Clontech). Following the generation of complementary DNA (cDNA), mRNA expression was assessed using SYBR Premix Ex TaqII kit (Clontech) with primers listed in Additional file 1: Table S2. For 2D pancreatic differentiation, *GAPDH* was utilized as the housekeeping gene (HKG). The expression was compared by assessing the fold-change relative to the HKG. For all other studies, 18S was used as the HKG. The expression was compared against a specified control group via the 2^−ddCt^ methodology.

### Single-cell RNA-sequencing of pancreatic progenitors

Cells were liberated from gel with 1 h 50 U/mL Type 1 collagenase treatment and dissociated with TrypLE Select for 15 min. Following, the cells were washed to remove debris and filtered with 40 µm strainer. Cells were washed three additional times with DPBS with 2%BSA (1000 rpm × 3 min). Approximately 10,000 single cells were recovered, and cDNA libraries were generated utilizing the v3.1 Next GEM Single Cell 3’ reagent kit and a Chromium single-cell system (10 × Genomics) and sequenced on a NovaSeq 6000 sequencer (Illumina) at the Center for Medical Genomics at Indiana University School of Medicine. Raw data were processed using the Cell Ranger pipeline. In brief, de-multiplex raw base sequence calls were aligned to human reference genome GRCh38 using RNAseq aligner Spliced Transcripts Alignment to a Reference (STAR). Aligned reads were traced back to individual cells, and gene expression in single cells was quantified using unique molecular identifiers (UMIs).

RStudio v2021.09.0 running R language v4.1.2 was used as the platform for the analysis of the scRNA-seq data of the merged dataset of 2D control and GelNB condition. The initial normalization, clustering, and finding differential expressed genes (DEGs) were performed by Seurat package v4.1.0 [21]. The matrix of read-count data of each cell for each gene was loaded individually and converted to Seurat objects. Cells containing extremely high percentage of mitochondrial gene counts, or cells containing extremely high or low housekeeping gene (RPL27) were removed from the pool. After applying merge function of Seurat to combine each dataset of 2D control and GelNB hydrogel, the gene expression levels were normalized by SCTransform function and variable gene identification was done at the same time. Dimension reduction was performed by the principal component analysis (PCA), and the number of the principal components was determined based on the elbow plot of the standard deviation explained by each principal component. Shared nearest neighbor graph construction and unsupervised clustering were performed, followed by finding differentially expressed genes (DEG) in each cluster for determination of cluster identity (Additional file 1: Table S3), and uniform manifold approximation and projection plot (UMAP) was calculated. FindMarkers function of Seurat was performed to find DEGs in the pancreatic progenitors between GelNB or 2D control, and the trophectoderm subsets in GelNB. The DEGs were used for volcano plots.

Pancreatic progenitors in 2D control and GelNB were processed by DESeq2 v1.34.0 [22], followed by Gene Set Enrichment Analysis (GSEA) using the Integrative Differential expression and gene set Enrichment Analysis (iDEA) v1.0.1. Gene sets used for GSEA were downloaded from the Molecular Signatures Database (MSigDB) (http://www.gsea-msigdb.org/gsea/msigdb/collections.jsp).

The scRNA-seq dataset is accessible in the Gene Expression Omnibus with accession code GSE229058. All source codes were uploaded to the GitHub repository (https://github.com/CHIENCHILIN/Arkenberg_et_al_PPdiff). DESeq2 and GSEA codes were run on high-throughput computing cluster and storage resources at Indiana University.

### Statistical analysis

Statistical analyses were conducted using either two-way ANOVA with a Bonferroni’s post hoc test or two-tailed t-test using GraphPad Prism 8 software. Experiments were conducted independently at least three times. Data presented were Mean ± SEM. Single, double, triple, and quadruple asterisks correspond to *p* < 0.05, 0.01, 0.001, and 0.0001, respectively. A *p* < 0.05 was considered statistically significant.

## Results

### Pancreatic differentiation of iPSCs in 3D led to prominent tubular network

To establish a benchmark of PP differentiation, ChiPSC12 cells were plated in a 6-well plate and maintained in Essential 8 (E8) media until reaching 90% confluency, at which point PP differentiation was initiated using STEMDiff^TM^ pancreatic progenitor kit. During differentiation, the expression of signature genes for pluripotency (*OCT4*, *NANOG*), DE (*SOX17*, *FOXA2*), and PP (*PDX1*, *SOX9, NKX6.1*, and pancreas associated transcription factor 1a (*PTF1A*)) at the mRNA levels was quantified by real-time quantitative PCR (qPCR). As expected, expression of the pluripotency genes *OCT4* and *NANOG* was high during proliferation stage (S0), decreased markedly after DE differentiation (S1), and was completely undetectable after PP differentiation (S4) (Fig. 1a). Furthermore, DE signature genes *SOX17* and *FOXA2* were only transiently detected after S1 (Fig. 1a). PP signature genes, including *PDX1*, *NKX6.1*, *SOX9*, and *PTF1A*, were detected in high levels only after S4 (Fig. 1b). Flow cytometry analysis of S4 cells revealed that over 40% of the differentiated cells co-expressed PDX1 and NKX6.1 (Fig. 1c). For *in situ* 3D PP differentiation, we synthesized GelNB with low NB substitution, enabling the crosslinking of cell-laden hydrogels with low shear moduli (G′ ~ 0.5 kPa or 1 kPa. Fig. 1d). Of note, ROCK inhibitor Y-27632 was added in the first 4 days to maintain cell viability, followed by 2 additional days of culture in the absence of Y-27632. The removal of Y-27632 after 4 days of culture did not reduce cell viability as virtually all aggregates formed in both hydrogels were stained green (Additional file 1: Fig. S1a, iPSC panel). Next, cell-laden hydrogels were moved to DE differentiation media for 2 additional days and the live/dead staining results demonstrated nearly 100% cell viability (Additional file 1: Fig. S1a, DE panel). Successful DE differentiation was confirmed by significant upregulation of *SOX17* and *FOXA2* expression (Additional file 1: Fig. S1b). After DE differentiation, prominent sprouting was observed from cell clusters, which was accompanied by the expression of EMT-related markers (e.g., downregulation of *CDH1* and upregulation of *CDH2*, *SNAI1*, and *VIM.* Additional file 1: Fig. S1c)*.*

Encouraged by the successful DE differentiation of iPSCs encapsulated in the soft 0.5 kPa GelNB hydrogels, we explored subsequent PP differentiation within hydrogels with different crosslinking density (i.e., 5 wt% GelNB with 0.6 wt% or 1 wt% PEGSH crosslinker for crosslinking into hydrogels with ~0.5 kPa or 1 kPa, respectively. Fig. 1d). iPSC-laden GelNB hydrogels were treated with the 4-stage PP differentiation kit. During the first two stages of PP differentiation (DE and PGT), we observed minimal morphological differences between the two hydrogel formulations (Fig. 1e). DE differentiation (S1) led to infilling at the center of the aggregates as well as microprotrusions formation in the 0.5 kPa hydrogels (Additional file 1: Fig. S1a). At the PGT stage (S2), notable protrusions started to form in 0.5 kPa hydrogels, whereas 1 kPa hydrogels appeared to suppress this morphology. PGT clusters formed in 0.5 kPa GelNB hydrogels began to form a thin epithelium-like layer with outward cell migration. At the PF stage (S3), 0.5 kPa hydrogels supported budding epithelial structures, whereas 1 kPa hydrogels led to both solid aggregates and thin, cystic structures. After 4-stage of PP differentiation, multi-cellular aggregates were notable in both 0.5 kPa and 1 kPa hydrogels but only 0.5 kPa hydrogels supported pronounced tubular network formation. To assess the efficiency of pancreatic differentiation in 3D hydrogels, we evaluated the expression of a panel of PP-associated markers through immunofluorescence staining and confocal imaging (Fig. 1f). We observed a high degree of endoderm-associated GATA4/SOX9 expression and the presence of few PDX1/NKX6.1-postive PP cells in both the 0.5 kPa and 1 kPa hydrogels.

### PP differentiation in 3D led to diverse cell populations

**Fig. 2:**
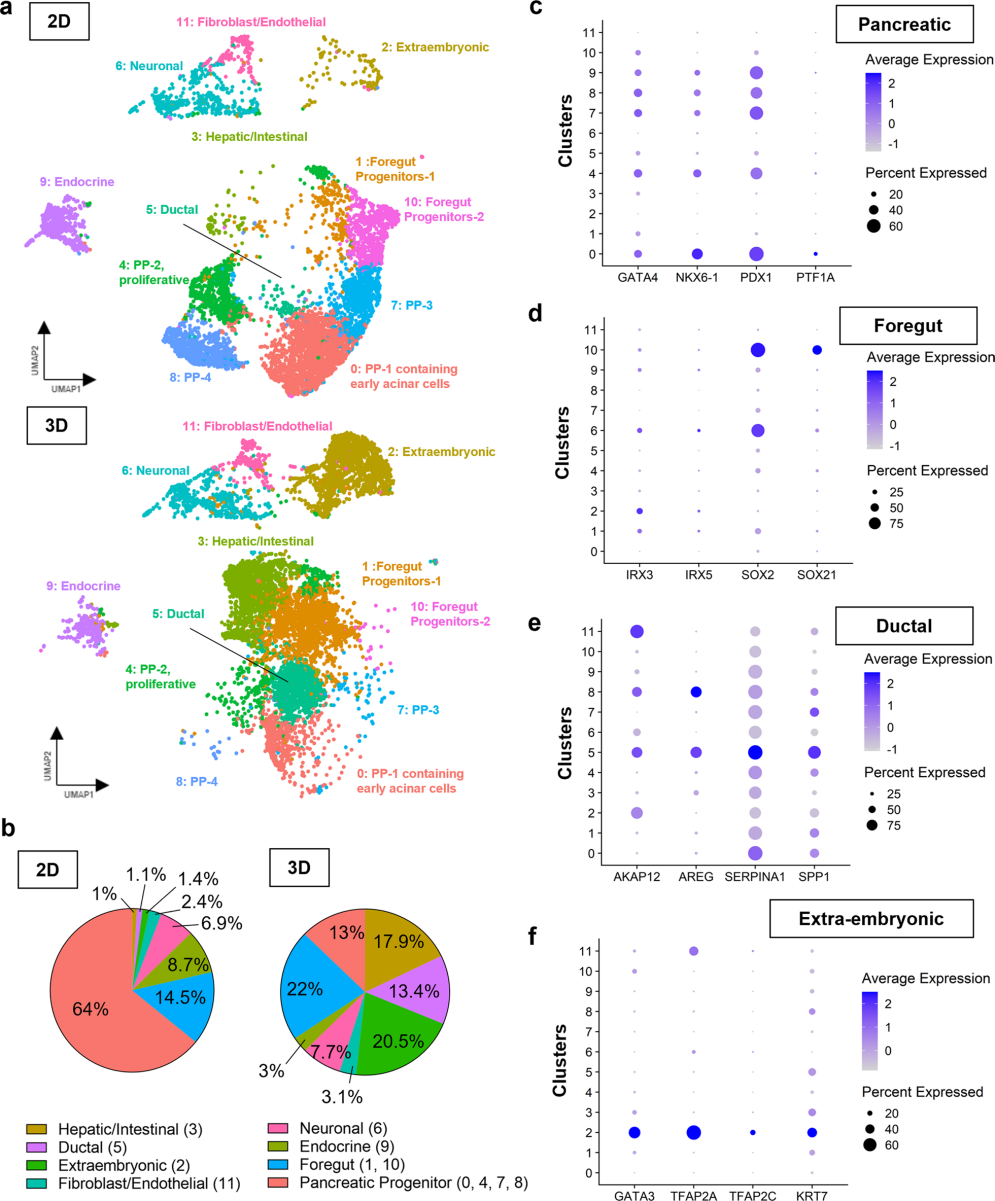
scRNA-seq analysis of PP-stage differentiating cells reveals emergence of extraembryonic cell populations in the hydrogels. **a** UMAP of scRNA-seq after 4-stage PP differentiation in 2D and 3D (0.5 kPa GelNB hydrogel) conditions. Data represent 7468 and 8447 total cells for 2D and 3D conditions, respectively. **b** Percentage of cell populations from scRNA-seq analysis in 2D and 3D conditions. **c**–**f** Dot plots of upregulated genes in pancreatic (**c**), ductal (**d**), foregut (**e**), and extraembryonic (**f**) clusters

To further assess the identities of the cell populations generated after the 4-stage PP differentiation, approximately 8,000 cells from 2D monolayer and 3D hydrogel differentiation were profiled using single-cell RNA-sequencing (scRNA-seq). Populations were annotated by manually assessing the expression of markers associated with the specified cells (Fig. 2a and b). The top 30 differentially expressed genes for all identified clusters are listed in Additional file 1: Table S1. In the 2D condition, ~64% of cells were identified as PP cells (combined clusters #0, #4, #7, and #8) for their expression of *GATA4*, *NKX6.1*, *PDX1* (Fig. 2c). Among the four pancreatic populations, cluster #0 (i.e., PP-1) exhibited significant expression of tip-associated pancreatic progenitor markers *DLK1*, *SPINK1*, *CLPS*, *CPA1*, and *CPA2* [23] (Additional file 1: Table S1). While cluster #4 (i.e., PP-2) also expressed similar pancreatic progenitor markers (e.g., *PDX1*, *NKX6.1*), the top differentially expressed genes were primarily cell cycle-associated including *TOP2A*, *CENPF*, *MKI67*, *UBE2C*, and *NUSAP1* among others [24], indicating that this cluster was highly proliferative. Cluster #1 (i.e., PP-1) differentially expressed genes associated with acinar lineages, whereas cluster #7 (i.e., PP-3) might be associated with endocrine/ductal differentiation with high expression of *NKX6.2*, *ONECUT1*, and *ANXA4* [25–27]. Interestingly, this population also expressed *CALB1* which is associated with the kidney duct [28]. Lastly, cluster #8 (i.e., PP-4) was enriched in inflammatory and EMT-inducing markers, often associated with pancreatitis and pancreatic ductal adenocarcinoma, such as *LTB* [29], *IL32* [30], and *AREG* [31]. Cluster #9 was assigned to endocrine cells for their high expression levels of *CHGA*, *INS*, *NEUROG3*, and *NKX2.2* [32] (~8.7%, cluster #9. Additional file 1: Fig. S2a). We also identified clusters #1 and #10 as a combination of anterior foregut progenitors (expressing *IRX3*, *IRX5*, *SOX2*, and *SOX21 *[33]) (Fig. 2d), as well as an uncharacterized endodermal progenitor population (~14.5%). In addition, cluster #5 (~1.1%) was characterized as duct-like cell expressing known small ductal markers, *AKAP12*, *AREG*, *SERPINA1*, and *SPP1* [34] (Fig. 2e), cluster #3 (1%) contained both early intestinal cells expressing *CDX2*, *HOXA9*, *HOXB6*, and *HOXB9* [33] (Additional file 1: Fig. S2b) and early hepatocytes expressing *ALB*, *CYP1A1*, *FGB*, and *TF* [35, 36] (Additional file 1: Fig. S2c). In addition to endoderm-related cell populations, we also identified a small population (~2.4%) of endothelial cells (Additional file 1: Fig. S2d) and fibroblasts (Additional file 1: Fig. S2e) for their expression of *CD34*, *CDH5*, *ESM1*, *KDR* [37] and *ACTA2*, *CD248*, *PDGFRA*, *LUM* [38], respectively. Approximately 6.9% of cells (cluster #3) were characterized as neuronal cells as indicated by *HES5*, *PANTR1*, *S100B*, and *STMN4* expression[39–42] (Additional file 1: Fig. S2f). Finally, one small cluster (#2, ~1.4%) was labeled as extraembryonic-like cells for their expression of *GATA3, TFAP2A, TFAP2C*, and *KRT7* [43] (Fig. 2f).

**Fig. 3:**
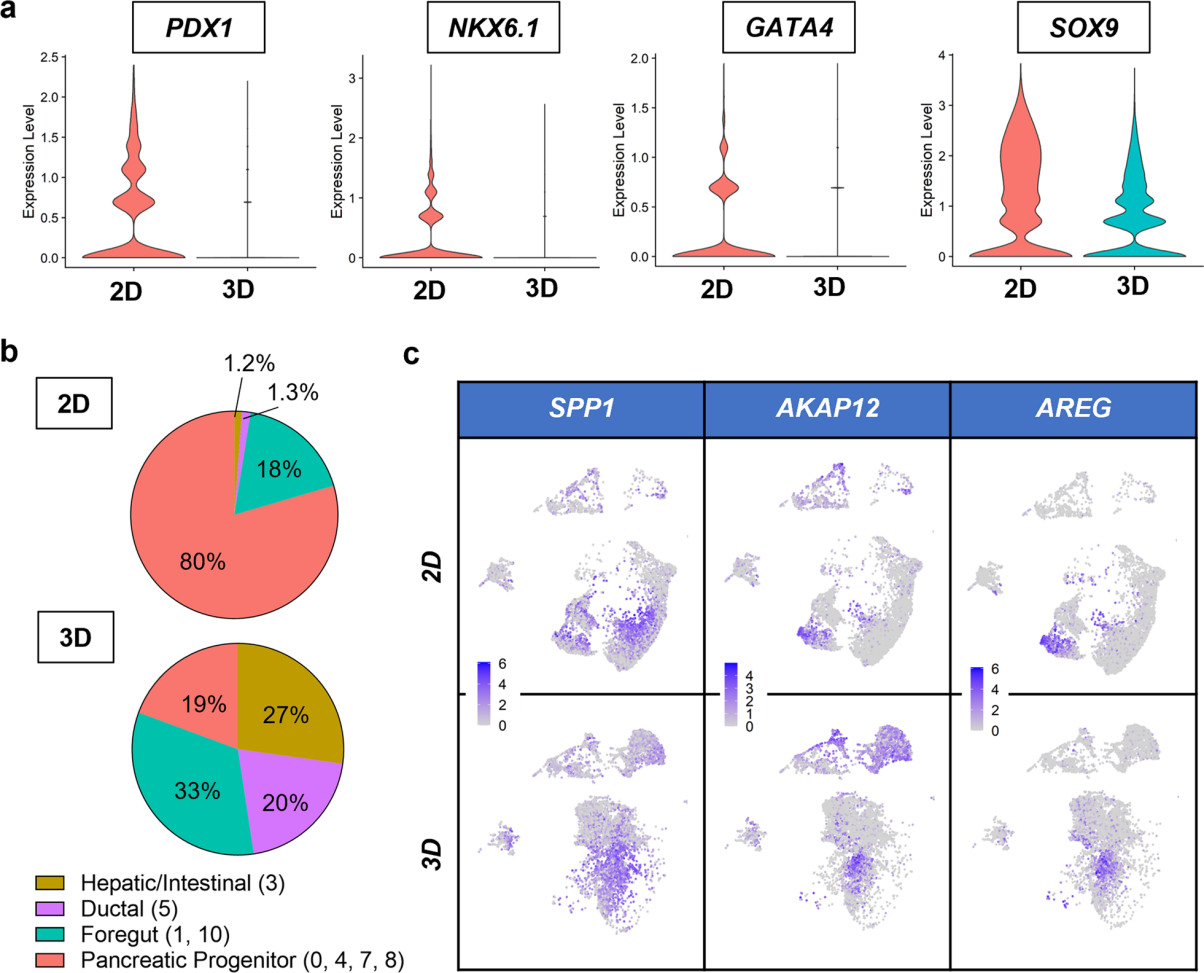
Differentiation in 3D hydrogels reduces pancreatic progenitor populations but enriches other endodermal cells. **a** Violin plots of pancreatic progenitor markers *PDX1*, *NKX6.1*, *GATA4*, and SOX9 following pancreatic progenitor differentiation in 2D and 3D conditions. **b** Percentage of endodermal cell populations from scRNA-seq analysis in 2D and 3D conditions. **c** Feature plots of ductal cell markers *SPP1*, *AKAP12*, and *AREG* in 2D and 3D conditions

After broadly annotating cell clusters within both 2D and 3D samples, we compared each condition with respect to endoderm-associated markers. Specifically, the combined percentage of the four pancreatic clusters were 67% and 13% in 2D and 3D, respectively (Fig. 2b). Global expression of endoderm markers *PDX1, NKX6.1, GATA4,* and *SOX9* was largely reduced when the cells were differentiated in 3D (Fig. 3a). Within the endoderm cell populations (clusters 0, 1, 3, 4, 5, 7, 8, 10, Fig. 3b), PP differentiation in 2D led to majority of PP cells (80%), followed by foregut cells (18%) and a small population of hepatic/intestinal and ductal cells (~2.5%). In contrast, the biggest population in the 3D differentiated cells was foregut progenitors (33%), followed by hepatic/intestinal-like cells (27%), ductal-like cells (20%), and PP cells (19%) (Fig. 3b). Within the ductal-like cell population (cluster 5), we observed a population of cells expressing *SPP1* and *AKAP12* (Fig. 3c), which have been shown to associate with the pancreatic pro-ductal genotypes [34]. Further, this population was also enriched with *AREG* expression (Fig. 3c), which might be implicated in EMT [31]. Finally, we observed a notable population of cells (~20.5%, cluster 2) with high levels of *GATA3, TFAP2A, TFAP2C,* and *KRT7* expression (Fig. 2a, b, f) that were typical of cells in the extraembryonic tissues.

### PPs generated in 3D hydrogels are genetically distinct from their 2D counterparts

**Fig. 4:**
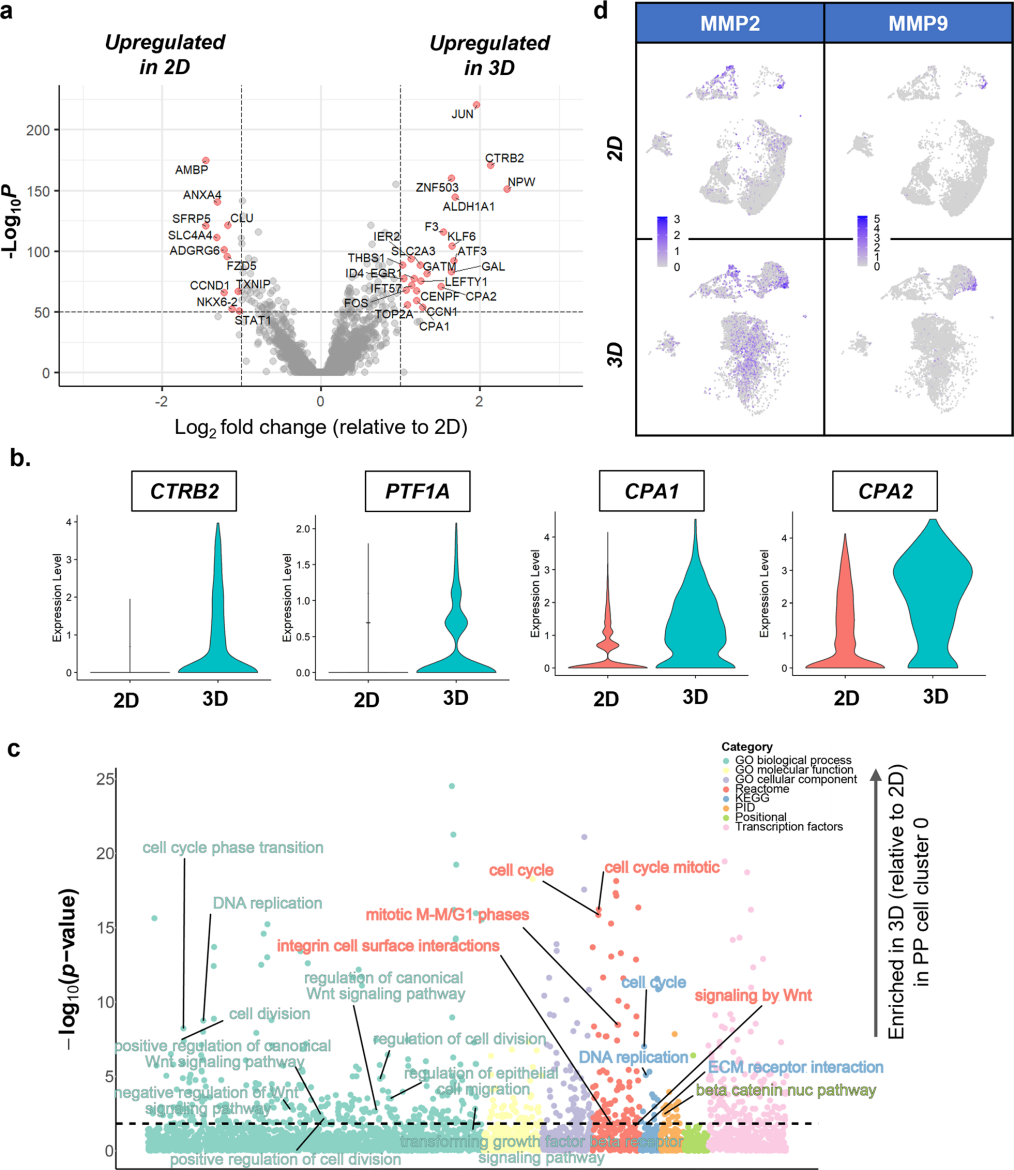
PPs in hydrogels are genetically distinct from 2D cultured counterparts. **a** Volcano plot showing enrichment of genes within cluster 0 in 2D (left) and GelNB (right). **b** Violin plots of pro-acinar marker expression in 2D and GelNB conditions. **c** Bubble plots of enriched gene sets within cluster 0 in GelNB relative to the 2D control. **d** Feature plots of MMP2 and MMP9 expression in 2D and GelNB conditions

Although we observed a lower prevalence of PP cells in 3D, we sought to compare differences in gene expression patterns within the PPs identified in 2D and 3D hydrogel conditions. Cluster #0 (i.e., PP-1) was chosen for comparisons between both groups given the high levels of PP marker expression (Fig. 2c) [23]. Relative to 2D culture, PP-1 cells generated in the hydrogels exhibited higher expression levels of pro-acinar (e.g., *CTRB2*, *CPA1,* and *CPA2*) [23, 44] and liver associated genes (e.g., *ALDH1A1* and *ATF3*) [45–47] (Fig. 4a and b). In addition to distinctions in cell identities, PP-1 cells in the 3D hydrogels were also enriched in genes associated with proliferation, including expression of *TOP2A*, *CENPF*, and *ZNF503* [24] (Fig. 4a). GSEA analysis comparing PP-1 cells within the 3D hydrogels relative to the 2D condition also showed differences in enrichment based on culture dimensionality. Specifically, pathways associated with cell division were enriched in 3D GelNB hydrogels (Fig. 4c). The enriched pathways include gene sets associated with cell division, cell cycle, and DNA replication. In addition, enriched pathways included canonical and noncanonical Wnt as well as TGFβ signaling. Differentiating cells in 3D hydrogels also led to distinct expression of MMPs, integrins, and ECM molecules. Specifically, the expression of gelatinases *MMP2* and *MMP9*, which aside from degrading gelatin, also degrades Type IV collagen[48, 49], was elevated in the 3D GelNB hydrogels than in 2D control (Fig. 4d). These MMPs were primarily localized to the fibroblast and extraembryonic populations but were also expressed in the pro-ductal cell population in 3D GelNB gels, especially for MMP2 expression.

### Extraembryonic cell populations were present after pancreatic differentiation in 3D but not 2D.

**Fig. 5:**
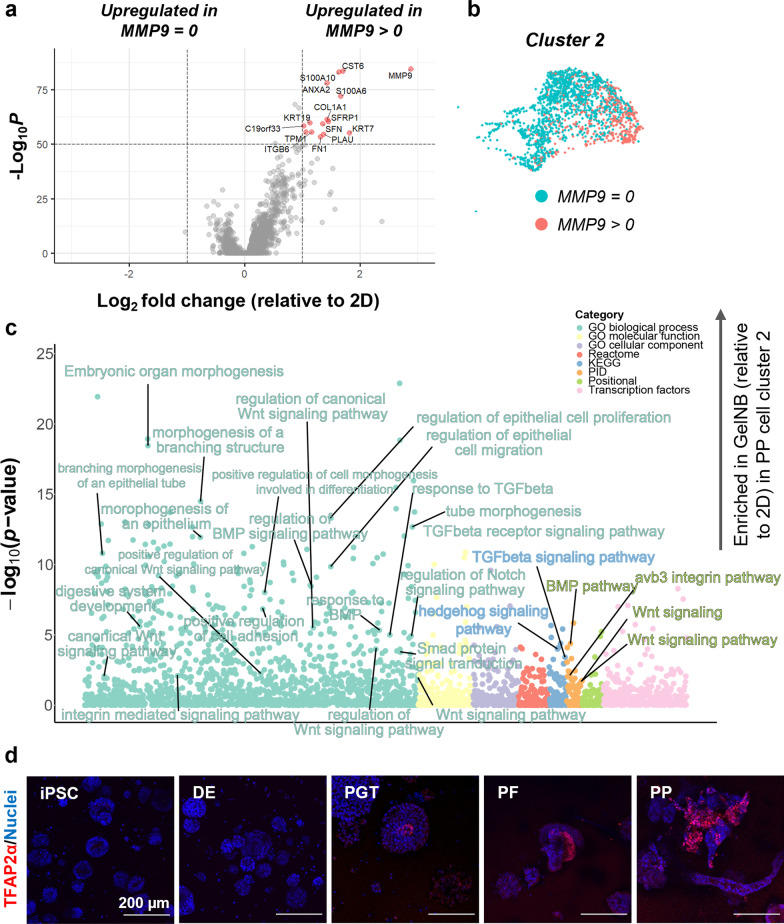
Emergence of extraembryonic cells in 3D hydrogels following PP differentiation. **a** Volcano plot showing enrichment of genes within cluster 2. **b** Feature plot of cluster 2 showing cells with MMP9 upregulation. **c** Bubble plots of enriched gene sets within cluster 2 in 3D relative to the 2D control. **d** Representative immunostaining images of TFAP2A-positive extraembryonic cells at each stage of differentiation in 3D GelNB hydrogels. At least 3 regions of interest were imaged (10 slices in each z-stack with a slice height of 10 µm)

In addition to the variation of PP/endoderm cell populations between 2 and 3D differentiation protocols, we observed a dramatically higher percentage (~ 20-fold increase) of cells harboring extraembryonic genes (cluster #2) in the 3D gels compared with the 2D control (Fig. 2a, b, f). This population exhibited expression of trophectoderm transcriptional regulators, *GATA3, TFAP2A, TFAP2C, and KRT7* [43] (Additional file 1: Figs. S3a, S3b). Interestingly, *GATA2*, another regulator of trophectoderm cell function [43] had minimal expression in all conditions (Additional file 1: Fig. S3a). We also assessed other known markers of trophectoderm cell populations including *TP63*, *CDH5*, *HLA-G*, *MMP9*, and *PLAU,* which were all significantly upregulated in the 3D hydrogels (Additional file 1: Fig. S3a), further confirming the identity of the trophectoderm cells emerged in the hydrogel samples after PP differentiation [50, 51]. Within the extraembryonic cells, we identified two subpopulations delineated by expression of *MMP9*. Interestingly, the *MMP9* subset was enriched in expression of other ‘typical’ trophectoderm markers, including *KRT7, PLAU, KRT19, S100A10, and S100A6* [52] (Fig. 5a, b). Examination of enriched gene sets comparing the extraembryonic populations in 2D and 3D samples revealed upregulation of a number of morphogenesis-specific pathways in the 3D GelNB condition (Fig. 5c). While the exact mechanisms by which 3D hydrogels generated extraembryonic cells from iPSCs are unknown and further studies are warranted, these trophectoderm-like cells expressed Wnts including *WNT3*, *WNT5A*, *WNT6*, and *WNT9B* as well as *BMP4*, *BMP5*, *BMP7*, and *GDF6* that might influence downstream differentiation of iPSCs into endoderm lineages (Additional file 1: Fig. S4a and S4b). Immunostaining of TFAP2A, a trophectoderm marker, revealed that the trophectoderm cells appeared at the center of the aggregate during PGT stage of differentiation and proliferated substantially in the subsequent stages (Fig. 5d).

### Inhibition of BMP and Wnt signaling enhanced PP cell differentiation in GelNB hydrogels

**Fig. 6:**
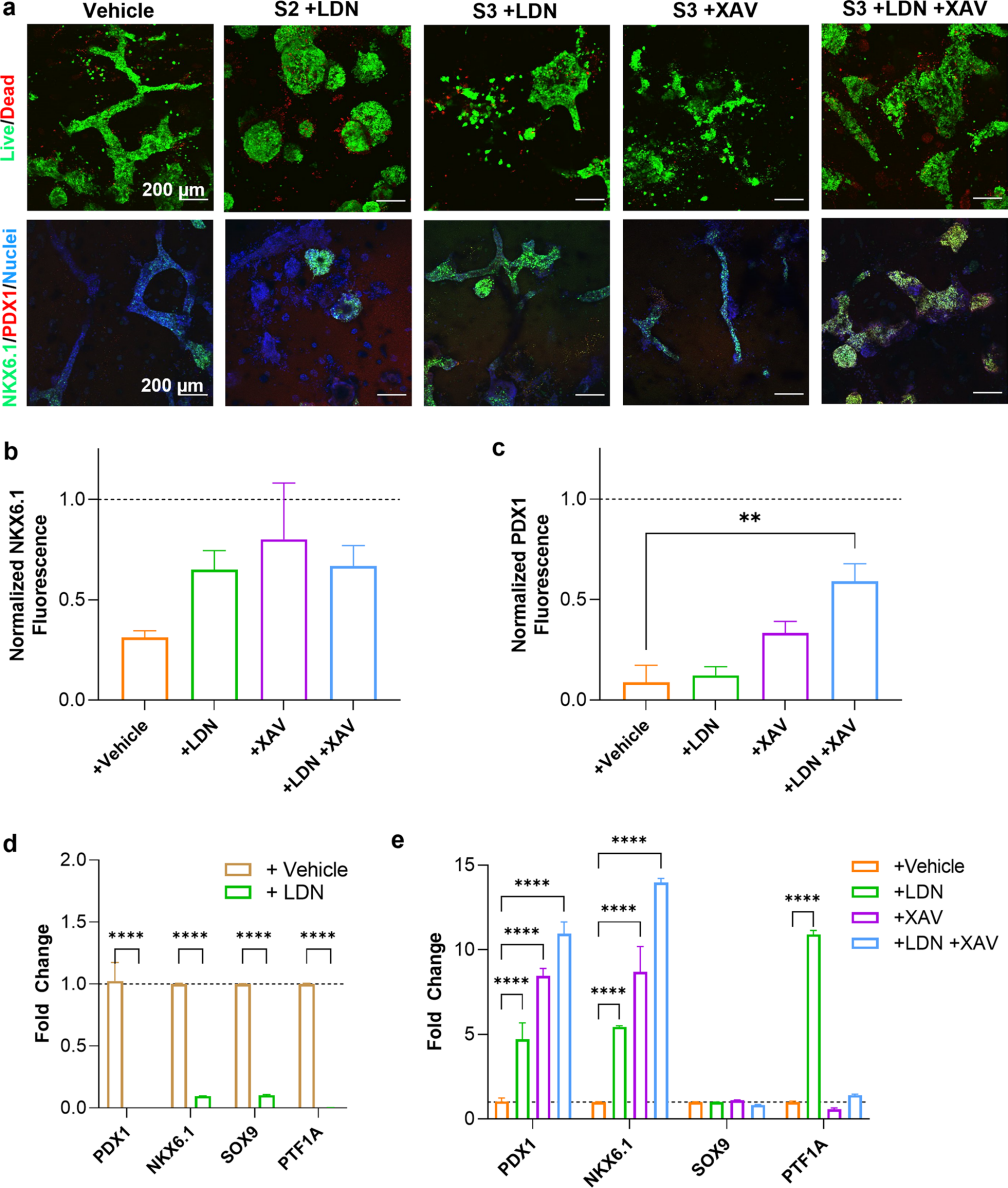
Small molecule inhibition of BMP and Wnt signaling pathways during pancreatic differentiation. **a** Representative live/dead and immunostained images of treated cells at the PP stage. Quantification of **b** NKX6.1 and **c** PDX1 fluorescence normalized to the nuclear stain in the immunostaining images. Statistical significance was determined by a one-way ANOVA relative to the vehicle control. (**p < 0.01, *N* = 3 images per condition). **d** *PDX1*/*NKX6.1*/*SOX9*/*PTF1A* mRNA expression with or without LDN treatment starting at the S2 stage. **e** *PDX1*/*NKX6.1*/*SOX9*/*PTF1A* mRNA expression with or without LDN/XAV treatment starting at the S3 stage. For panels **d** and **e**, significance was determined via two-way ANOVA relative to the vehicle control. (**** represents *p* < 0.0001, *N* = 3 samples per condition)

We hypothesized that elevated endogenous Wnt and BMP expression from cells within the 3D samples may be hindering pancreatic differentiation; therefore, we employed BMP inhibitor LDN193189 and Wnt inhibitor XAV939 to assess the influence of pathway inhibition on PP differentiation outcome, with DMSO added as a vehicle control. Consistent with prior results, cells in the vehicle control group formed pronounced tubular structure after 4-stage PP differentiation, but few cells were positive for PP markers NKX6.1 and PDX1 (Vehicle, Fig. 6a). Addition of BMP inhibitor LDN193189 starting either from Stage 2 (i.e., following DE stage) or Stage 3 (i.e., after PGT stage) did not affect cell viability but led to large cell spheroids rather than the branched epithelium observed in the vehicle control group (S2+LDN & S3+LDN. Fig. 6a). BMP inhibition also reduced the number of PP cells as indicated by immunostaining and imaging (Fig. 6a) and qPCR results (Fig. 6d). On the other hand, addition of Wnt inhibitor XAV939 starting from the PGT stage (S2) led to widespread cell death (Additional file 1: Fig. S5), We also treated the cells with the inhibitors at the start of the PF stage and identified differences in aggregate morphology and PP cell fate. We observed more ductal morphologies with some rounded aggregates in the BMP-inhibited condition (S3+LDN, Fig. 6a), thin epithelial structures in the Wnt-inhibited condition (S3+XAV, Fig. 6a), and both rounded and duct epithelium morphologies in the dual inhibited condition (S3+LDN+XAV, Fig. 6a). Critically, inhibition of Wnt signaling at the PF stage did not significantly reduce the cell viability but promoted PP cell identity with a higher number of PDX1+/NKX6.1+ progenitors (Fig. 6b and c) relative to the vehicle control. This was confirmed by qPCR, with ~10-fold and ~14-fold increase in *PDX1* and *NKX6.1* expression, respectively (Fig. 6e). Interestingly, minimal difference in *SOX9* expression was observed; however, a ~10-fold increase in pro-acinar marker *PTF1A* was measured in the BMP inhibitor treated sample alone, suggesting that BMP signaling may be implicated in reducing the pro-acinar PP cell differentiation.

## Discussion

ChiPSC12, an iPSC line derived from skin fibroblasts of a 24-year-old healthy adult male of European descent, was chosen as it has been successfully differentiated into pancreatic β-cells in conventional 2D mono-layer culture and in 3D spheroids [53]. We first verified pancreatic differentiation in 2D with the ChiPSC12 cell line using a commercially available kit (Fig. 1a–c). As expected, stage-specific markers were present with corresponding stages of differentiation. We have shown that matrices and substrates with moderately high stiffness (e.g., G′ ~ 1.5 kPa) supported DE differentiation from iPSCs [17]. It was not clear; however, whether the DE cells could be further differentiated into PP cells in 3D. As higher matrix stiffness led to low cell viability, we reasoned that softer hydrogels might facilitate cell proliferation and morphogenesis [54, 55]. However, it was not clear if chemically crosslinked hydrogels with softer mechanics (e.g., G′ < 1 kPa) could also support DE differentiation. Here, we show that the 0.5 kPa hydrogels supported DE differentiation (Additional file 1: Fig. S1). In contrast to the 1.5 kPa gels, the softer hydrogels permitted formation of protrusions resembling migratory phenotypes with elevated expression of EMT markers. This EMT phenotype was consistent with observations in the literature [56–58]. Following assessment of DE differentiation in the 0.5 kPa GelNB hydrogels, we monitored the morphology of the aggregates at various stages of differentiation (Fig. 1e). Interestingly, in these mostly elastic GelNB hydrogels, we observed hollow and cystic iPSC aggregates after 4-days of culture, which were similar to that reported using fast stress-relaxing alginate hydrogels [17, 59]. This suggests that protease degradable hydrogels (e.g., GelNB) with soft mechanics might be sufficient to induce the formation of polarized aggregates for pancreatic differentiation. Continued observation of morphological changes at each stage of differentiation revealed significant migration away from the core of the aggregates at the PGT stage and initial epithelialization and budding in the PF stage. At the PP stage, a plexus of branched tubules was prevalent in the 0.5 kPa gels. These stage-specific morphological differences appeared to resemble early pancreatic organogenesis in vivo, which was characterized by the appearance of a microlumenal network that mature into acinar and ductal networks [60, 61]. It has been shown that the pancreatic endoderm interacts with the matrix through integrin signaling, and this interactions may also be influencing polarization and branching of the endoderm epithelium [60, 62]. This process appeared to be mechanically dependent, as the stiffer (G′ ~ 1 kPa) hydrogels restricted the branching development, which may be a prerequisite for pancreatic duct formation [63]. While morphological distinctions were clear, an assessment of the molecular identity of the cells was necessary to confirm the presence of the target pancreatic cells. Interestingly, we observed general expression of pan-endoderm markers GATA4 and SOX9, but the expression of pancreatic progenitor markers PDX1 and NKX6.1 was not detected in high levels regardless of matrix stiffness. This suggests that the tubular/ductal structures observed in the 3D GelNB hydrogels may not be pancreatic progenitors.

To identify cell populations within our differentiated samples, we conducted scRNA-seq on both the 2D and 3D-treated cells (Figs. 2 and 3). Consistent with our immunostaining results, compared with 2D samples, differentiation in 3D hydrogels led to significantly lower percentages of pancreatic progenitor populations, including clusters #0 (PP-1), #4 (PP-2), 7 (PP-3), and #8 (PP-4) and elevated levels of other endoderm and non-endoderm cells. Uniquely, we identified a population of ductal cell expressing *SPP1, AKAP12,* and *AREG* (Fig. 3c)*.* Upregulation of *AREG* was shown to increase EGFR/ERK signaling to drive cell migration and invasion [31]. We speculate that this population may be associated with the branched network at the PP stage within the 3D samples; however, additional investigation is warranted. Together, these data suggest that pancreatic differentiation of iPSCs within 3D hydrogels led to fewer PP cells but resulted in higher proportions of diverse endodermal (e.g., pro-hepatic and pro-intestinal cells), extraembryonic lineages, and potentially pro-ductal cells exhibiting migratory and assembly characteristics. The pronounced tubular network in 0.5 kPa hydrogels accompanied with reduction in PP cell populations, enrichment of other endodermal cells, and the emergence of extraembryonic cell populations indicated a crucial role of 3D microenvironment in stem cell fate determination. Beyond the optimization of soluble factors, further investigations are warranted for in situ 3D generation of pancreatic cells.

While notably less efficient, we did observe pancreatic progenitor populations within the hydrogels. To probe the differences between progenitors in 2D and 3D, we assessed differentiation expressed genes and employed the use of the iDEA pipeline to identify enriched gene sets within PP-1 cells in the two conditions (Fig. 4a–c). Interestingly, cells within the 3D gels were not only enriched in cell cycle-specific pathways but also pathways associated with both canonical and non-canonical Wnt signaling pathways [59], which are implicated in multiple morphogenetic processes of pancreatic development such as specification of the pancreatic endoderm, pancreatic bud formation, and epithelial cell expansion [64]. In addition, PP-1 cells in 3D hydrogels were enriched in ECM receptor and integrin cell surface interactions. While PP-1 cells in all conditions express traditional PP markers, *PDX1/NKX6.1*, given the enrichment in proliferation-associated gene enrichment pathways, we suspect the PPs in the 3D hydrogels may be less “mature” than those identified in 2D, potentially between the posterior foregut and PP stages. We suspect that the hydrogels may be ‘delaying’ the development and maturation of PPs, leaving cells in a cycling and more progenitor state.

Given the increased prevalence of extraembryonic cells, we conducted a similar assessment of gene expression and gene set enrichment comparing the extraembryonic cells in 2D and 3D (Fig. 5). Interestingly, we observed an *MMP9*+ subset resembling ‘typical’ extraembryonic cells [52]; however, all cluster 2 cells expressed canonical markers *GATA3, TFAP2A*, and *KRT7* (Fig. 5a and b)*.* Upregulated pathways in the cluster 2 cells in 3D compared to 2D were associated with Wnt, BMP, Notch, TGF-β, and hedgehog signaling, as well as morphogenesis of branching, tubular structures (Fig. 5c). This is consistent with the role of the trophectoderm layer in developmental processes, as the trophectoderm interacts directly with the endometrium and facilitates uterine invasion [65, 66]. In particular, Wnt signaling plays a critical role in the generation of CDX2+ intestinal/duodenal lineages, but requires repression for pancreatic and liver development [67, 68]. Further, BMP inhibition via small molecule treatment was shown to enhance pancreatic progenitor differentiation [67]. The extraembryonic cells were observed at the PGT stage and appeared proliferative as indicated by the increased number of TFAP2A+ cells in the gels (Fig. 5d). Additionally, these cells did not appear to assemble into the tubular structures, but rather spread loosely as mesenchymal-like cells. We speculated that the initial stage of iPSC proliferation into the cystic aggregates may be reverting the conventional, primed state iPSCs into a more naïve stem cell state [69] as it has been shown elsewhere that naïve stem cells exhibit greater propensity for trophectoderm stem cell differentiation [69]. However, future studies are needed to verify this hypothesis.

As mentioned above, we speculated that BMP and Wnt in the 3D hydrogels may be driving the duct-like and budding morphology and repressing pancreatic progenitor fate. Therefore, we hypothesized that inhibiting Wnts and BMPs at the right stage may reduce the morphogenic changes in the differentiating clusters and drive the differentiating cells toward a PP cell fate (Fig. 6). We initially attempted to inhibit Wnt and BMP at the PGT stage; however, a notable reduction in viability was observed in the Wnt-inhibited condition (Additional file: Fig. S5). This suggests that Wnt signaling is critical at early stages of pancreatic development to support cell viability. Interestingly, inhibition of BMP alone yielded high expression of PTF1A, an acinar cell marker. Previously, Haller *et al.* established a 4-stage differentiation protocol for generating pancreatic endocrine progenitors from iPSCs [70]. Noggin, a broad BMP inhibitor, was added in stage 3 (PGT) and 4 (PF), suggesting that BMP signaling is not desired for pancreatic progenitor differentiation. Regardless, inhibition of both BMP and Wnt signaling starting at the PF stage yielded highly elevated expression of pancreatic progenitor markers. While BMP inhibitor was likely added in the stages 3 and 4 supplements in the PP differentiation kit, the emergence of extraembryonic cells may have produced additional diffusible BMP and Wnt ligands that necessitate the addition of extra BMP/Wnt inhibitors for the desired PP differentiation. Taken together, these results confirm that the elevated gene expression of Wnts and BMPs, revealed by the RNA-seq results, influenced downstream assembly of complex epithelium structures as well as pancreatic fate within the 3D GelNB hydrogels.

## Conclusions

In conclusion, we utilized thiol-norbornene orthogonal photo-click chemistry to construct hydrogels for evaluating the effect of 3D hydrogel properties on pancreatic differentiation of human iPSCs. After confirming the utility of a commercially available kit for differentiation of ChiPSC12 cell line into primarily PP cells, we investigated the effect of hydrogel crosslinking density on the expression of pancreatic markers and multi-stage morphogenesis. Differentiation into DE cells was confirmed in ~ 0.5 kPa hydrogels and was accompanied by elevated EMT response. Utilizing single-cell transcriptomics, we determined that aggregate formation in hydrogels led to off-target endodermal and extraembryonic differentiation, as well as unique cell-ECM interactions. Differentiating cells within hydrogels were enriched in genes associated with BMP and Wnt signaling, and upon suppression of these pathways at the PGT stage, PP cell populations were greatly increased. Future work will include using engineered hydrogels for further characterization of the branched epithelial structures, enhancing pancreatic differentiation efficiency by identifying strategies to suppress or promote trophectoderm-like cell differentiation, and additional differentiation of the cells into maturated exocrine and endocrine organoids.

### Supplementary Information

**Additional file 1. **Supplementary tables and figures.

## Data Availability

The scRNA-seq dataset is accessible in the Gene Expression Omnibus with accession code GSE229058 at https://www.ncbi.nlm.nih.gov/geo/query/acc.cgi?acc=GSE229058. All other datasets used and/or analyzed during the current study are available from the corresponding author on reasonable request.

## Abbreviations

iPSCs: Induced pluripotent stem cells
2D: Two-dimensional
3D: Three-dimensional
PP: Pancreatic progenitor
DE: Definitive endoderm
TGF-β: Transforming growth factor beta
PGT: Primitive gut tube
PF: Posterior foregut
PDX1: Pancreatic and duodenal homeobox 1
NKX6.1: NKX6 homeobox 1
ECM: Extracellular matrix
ESC: Embryonic stem cells
dECM: Decellularized ECM
PEGNB: Poly(ethylene glycol)-norbornene
GelNB: Gelatin-norbornene
EDC: 1-(3-Dimethylaminopropyl)-3-ethylcarbodiimide
NHS: *N*-hydroxysuccinimide
LAP: Lithium aryl phosphinate
PEGSH: 4-Arm poly(ethylene glycol) thiol
DPBS: Dulbecco's phosphate buffered saline
VTN: Vitronectin
PFA: Paraformaldehyde
BSA: Bovine serum albumin
RT: Reverse transcription
qPCR: Quantitative PCR
HKG: Housekeeping gene
STAR: Spliced Transcripts Alignment to Reference
DEGs: Differential expressed genes
PCA: Principle component analysis
UMAP: Uniform manifest approximation and projection plot
GSEA: Gene set enrichment analysis
iDEA: Integrative differential expression and gene set enrichment analysis
MSigDB: Molecular Signatures Database
