# Supplementary material for: Photo-click hydrogels for 3D in situ differentiation of pancreatic progenitors from induced pluripotent stem cells
Source: Stem Cell Res Ther. 2023 Aug 30;14:223. doi: 10.1186/s13287-023-03457-7 (PMC10469883; doi:10.1186/s13287-023-03457-7)
Supplement: Supplementary file 1 — Additional file 1. Supplementary tables and figures. [file 13287_2023_3457_MOESM1_ESM.docx]

**Photo-click hydrogels for 3D *in situ* differentiation of pancreatic progenitors from induced pluripotent stem cells**

Matthew R. Arkenberg^1^, Yoshitomo Ueda^2^, Eri Hashino ^2,3^ and Chien-Chi Lin^1,4,5,^*

^1^Weldon School of Biomedical Engineering, Purdue University, West Lafayette, IN 47907, USA

^2^Department of Otolaryngology-Head and Neck Surgery, Indiana University School of Medicine, Indianapolis, IN 46202, USA

^3^Stark Neurosciences Research Institute, Indiana University School of Medicine, Indianapolis, IN 46202, USA

^4^Department of Biomedical Engineering, Indiana University-Purdue University Indianapolis, Indianapolis, IN 46202, USA

^5^Indiana University Simon Comprehensive Cancer Center, Indianapolis, IN 46202, USA

(Supporting Information)

*To whom correspondence should be sent:

Chien-Chi Lin, PhD.

Thomas J. Linnemeier Guidant Foundation Endowed Chair & Professor

Department of Biomedical Engineering

Purdue School of Engineering & Technology

Indiana University-Purdue University Indianapolis

723 W. Michigan St. SL220K

Indianapolis, IN 46202, USA

Phone: (317) 274-0760

Email: [lincc@iupui.edu](mailto:lincc@iupui.edu)


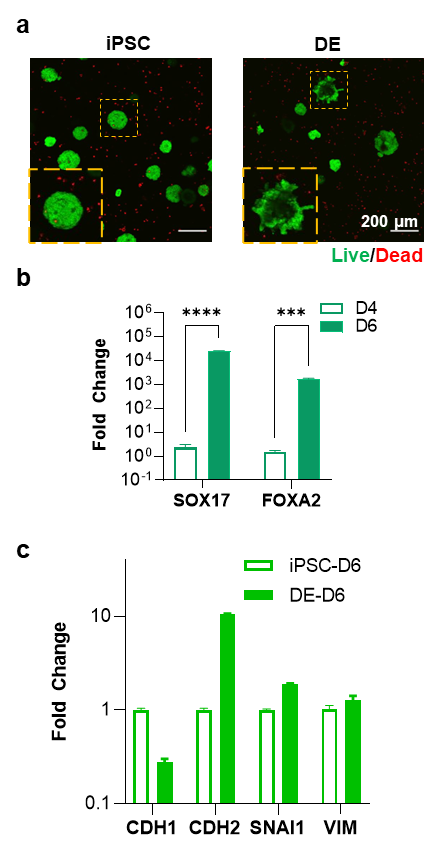


**Figure S1.** (a) Representative z-stacked images of live/dead stained cell-laden hydrogels. At least 3 regions of interest were imaged (10 slices in each z-stack with a slice height of 10 µm) (b) *SOX17*/*FOXA2* mRNA expression levels pre- and post-DE differentiation in hydrogels. (c) EMT-associated mRNA expression including *CDH1, CDH2, SNAI1, and VIM* in the IPSCs and DE differentiated samples. Unpaired t-tests were utilized to assess statistical significance (**, ***, and **** represent p-value < 0.01, 0.001, and 0.0001, respectively, N = 3 samples per condition).


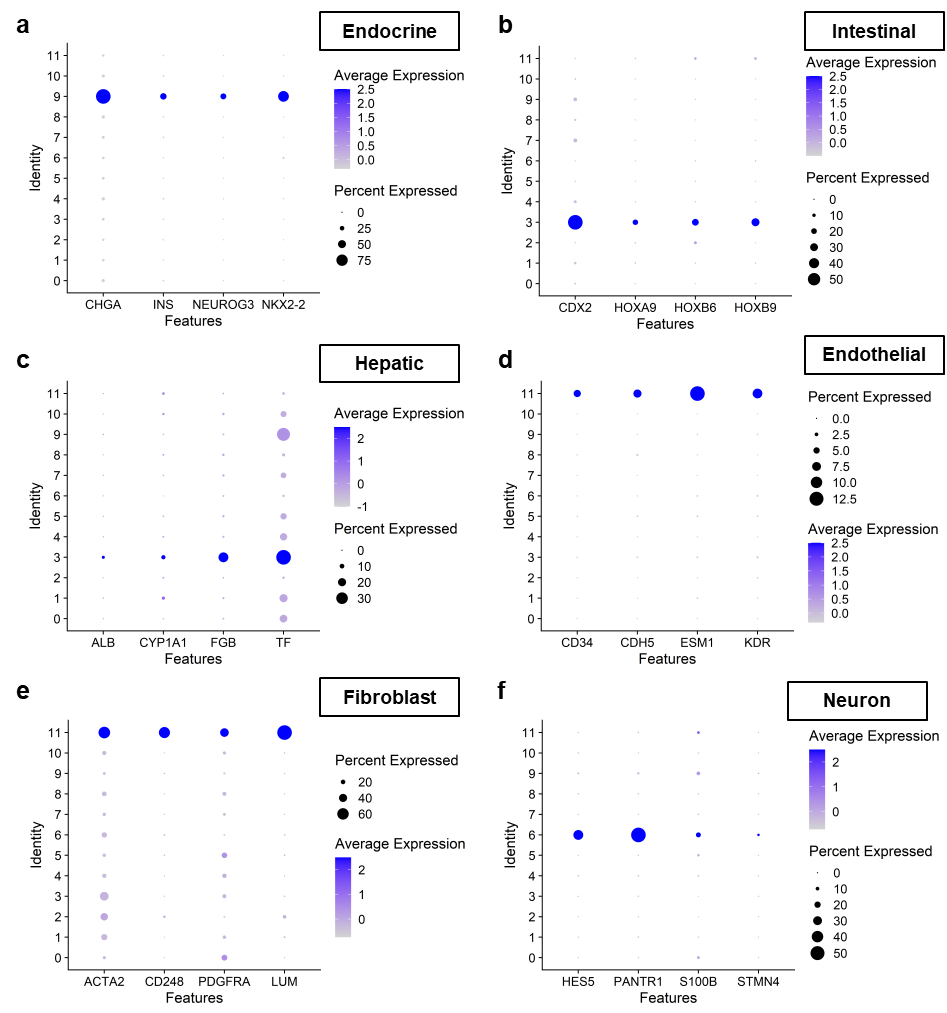


**Figure S2.** Dot plots of endoderm-associated features for (a) endocrine, (b) intestinal, (c) hepatic cells populations, as well as non-endoderm-associated features for (d) endothelial, (e) fibroblast, and (f) neuron.


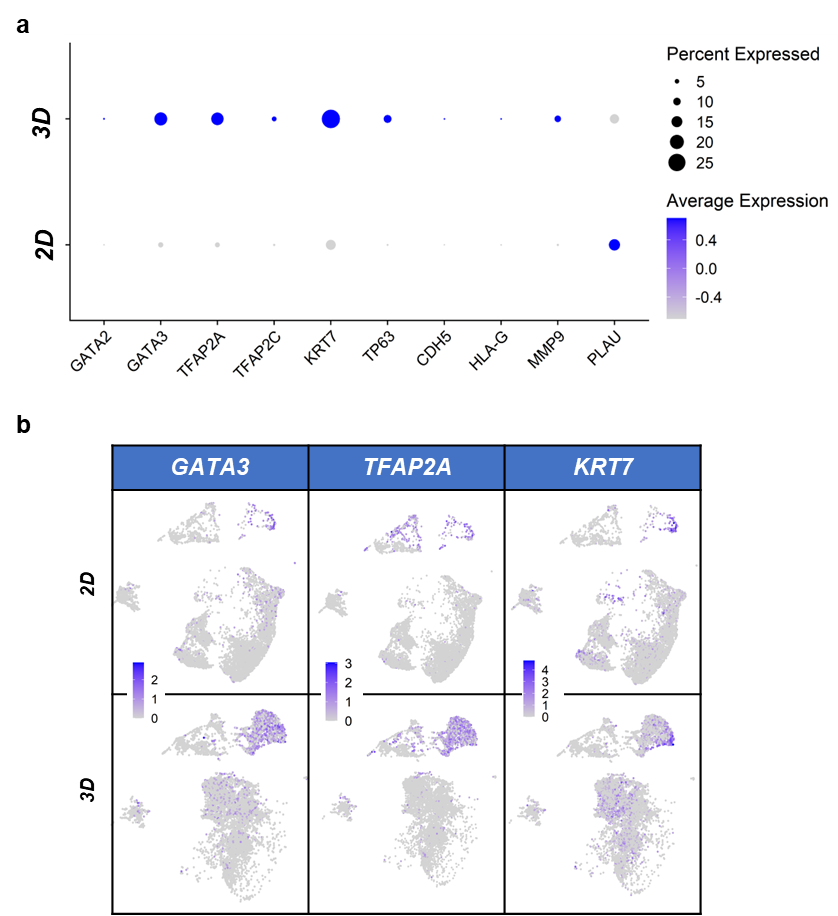


**Figure S3.** (a) Dot plots of common extra-embryonic markers *and GATA3*. (b) Feature plots of *GATA3, TFAP2A,* and *KRT7* expression in 2D and 3D conditions.


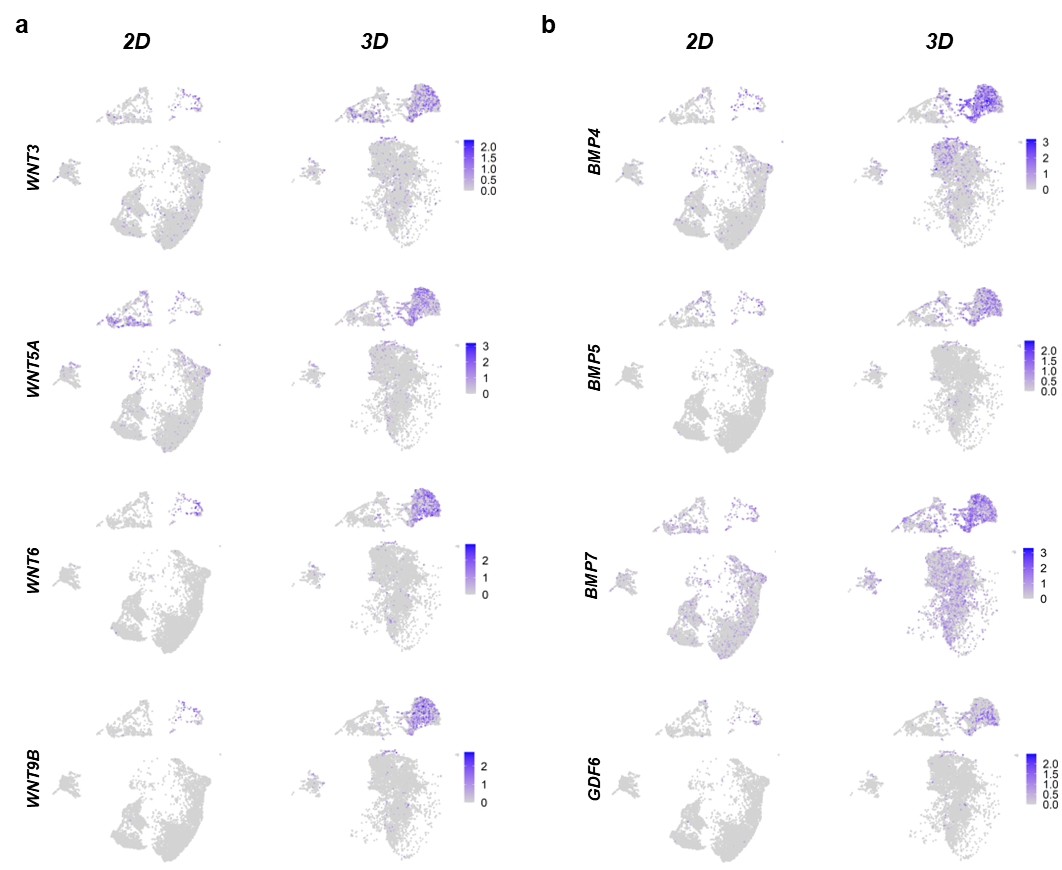


**Figure S4.** Feature plots of expressed (a) Wnts and (b) BMPs in 2D and 3D conditions.


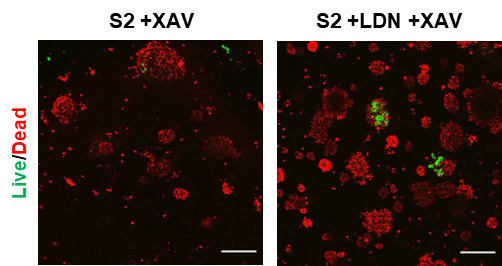


**Figure S5.** Live/dead staining and imaging of XAV and LDN/XAV treated differentiating iPSCs starting at stage 2 of differentiation.

**Table S1.** List of antibodies used in studies.

| Primary antibodies | Supplier | Species | Type | Dilution |
| --- | --- | --- | --- | --- |
| NKX6.1 | Cell Signaling | Rabbit | Monoclonal | 1:200 |
| PDX1 | Santa Cruz | Mouse | Monoclonal | 1:50 |
| GATA4 | Cell Signaling | Rabbit | Monoclonal | 1:200 |
| SOX9 | Santa Cruz | Mouse | Monoclonal | 1:100 |
| TFAP2A | Santa Cruz | Mouse | Monoclonal | 1:50 |
| Secondary antibodies |  |  |  |  |
| Anti-rabbit Alexa Fluor 488 | Thermo Scientific | Donkey | Polyclonal | 1:200 |
| Anti-mouse Alexa Fluor 647 | Thermo Scientific | Goat | Polyclonal | 1:200 |
| Anti-goat CruzFluor 647 | Santa Cruz | Mouse | Monoclonal | 1:200 |

**Table S2.** List of primers used in studies.

| Gene | Forward (5’->3’) | Reverse (5’->3’) | Ref |
| --- | --- | --- | --- |
| 18S | ATCCCTGAAAAGTTCCAGCA | CCTCTTGGTGAGGTCAATG | ^1^ |
| SOX17 | GTGGACCGCACGGAATTTG | GGAGATTCACACCGGAGTCA | ^2^ |
| FOXA2 | GGAGCAGCTACTATGCAGAGC | CGTGTTCATGCCGTTCATCC | ^2^ |
| CDH1 | GAAAGCGGCTGATACTGACC | GAAAGCGGCTGATACTGACC | ^3^ |
| CDH2 | TGTTTGACTATGAAGGCAGTGG | TCAGTCATCACCTCCACCAT | ^3^ |
| SNAI1 | GAGGCGGTGGCAGACTAG | GACACATCGGTCAGACCAG | ^4^ |
| VIM | GAACGCCAGATGCGTGAAATG | CCAGAGGGAGTGAATCCAGATTA | ^5^ |
| PDX1 | CGTCCGCTTGTTCTCCTC | CCTTTCCCATGGATGAAGTC | ^6^ |
| NKX6.1 | CTTCTGGCCCGGAGTGATG | GGGTCTGGTGTGTTTTCTCTTC | ^7^ |
| SOX9 | AGCGAACGCACATCAAGAC | CTGTAGGCGATCTGTTGGGG | ^8^ |
| PTF1A | AGAGAGTGTCCTGCTAGGGG | CCAGAAGGTCATCATCTGCC | ^9^ |

**Table S3.** List of top 30 DEGs in each cluster.

| **cluster** | **gene** | **pct.1** | **pct.2** | **p_val_adj** | **avg_log2FC** |
| --- | --- | --- | --- | --- | --- |
| 0 | SPINK1 | 1 | 0.9 | 0 | 2.520169 |
| 0 | CPA2 | 0.599 | 0.112 | 0 | 2.403104 |
| 0 | DLK1 | 1 | 0.809 | 0 | 2.310845 |
| 0 | IGF2 | 1 | 0.711 | 0 | 2.014122 |
| 0 | MEST | 0.884 | 0.678 | 0 | 1.573631 |
| 0 | AMBP | 0.988 | 0.665 | 0 | 1.503813 |
| 0 | H19 | 0.626 | 0.438 | 6.30E-141 | 1.483958 |
| 0 | CLPS | 0.116 | 0.024 | 1.20E-109 | 1.457765 |
| 0 | RTL1 | 0.641 | 0.137 | 0 | 1.449048 |
| 0 | SERPINA1 | 1 | 0.91 | 0 | 1.446966 |
| 0 | CPA1 | 0.47 | 0.073 | 0 | 1.369801 |
| 0 | STAT1 | 0.84 | 0.536 | 0 | 1.252174 |
| 0 | ONECUT1 | 0.963 | 0.557 | 0 | 1.251947 |
| 0 | SERPINA3 | 0.593 | 0.209 | 0 | 1.207238 |
| 0 | SOX9 | 0.907 | 0.608 | 0 | 1.177684 |
| 0 | TM4SF4 | 0.778 | 0.328 | 0 | 1.172812 |
| 0 | SERPINA5 | 0.841 | 0.404 | 0 | 1.166969 |
| 0 | CLU | 0.982 | 0.819 | 0 | 1.087501 |
| 0 | SLC4A4 | 0.808 | 0.438 | 0 | 1.079502 |
| 0 | MEG3 | 0.581 | 0.16 | 0 | 1.068314 |
| 0 | SFRP5 | 0.649 | 0.244 | 0 | 1.053501 |
| 0 | AUXG01000058.1 | 0.539 | 0.323 | 1.56E-141 | 1.034765 |
| 0 | LEFTY1 | 0.4 | 0.144 | 2.11E-217 | 0.938402 |
| 0 | CLMN | 0.67 | 0.228 | 0 | 0.931646 |
| 0 | FLRT2 | 0.948 | 0.632 | 0 | 0.931559 |
| 0 | CCND1 | 0.978 | 0.916 | 2.45E-229 | 0.929242 |
| 0 | MTUS1 | 0.835 | 0.525 | 0 | 0.921436 |
| 0 | GATM | 0.58 | 0.285 | 1.37E-235 | 0.911363 |
| 0 | LAPTM4B | 1 | 0.984 | 0 | 0.898356 |
| 0 | F3 | 0.487 | 0.221 | 5.75E-203 | 0.884992 |
| 1 | THBS1 | 0.787 | 0.271 | 0 | 2.011831 |
| 1 | PLA2G2A | 0.383 | 0.088 | 0 | 1.68496 |
| 1 | FST | 0.704 | 0.315 | 0 | 1.391653 |
| 1 | EDN1 | 0.484 | 0.191 | 4.76E-213 | 1.343089 |
| 1 | NUDT4 | 0.862 | 0.564 | 6.12E-293 | 1.065797 |
| 1 | RGS5 | 0.601 | 0.199 | 0 | 1.05972 |
| 1 | DKK1 | 0.347 | 0.078 | 2.78E-293 | 1.025131 |
| 1 | TTR | 0.743 | 0.405 | 6.04E-297 | 1.006702 |
| 1 | C1GALT1 | 0.836 | 0.548 | 6.31E-301 | 0.989206 |
| 1 | PRSS2 | 0.607 | 0.284 | 5.74E-203 | 0.968094 |
| 1 | JUN | 0.91 | 0.62 | 2.84E-278 | 0.968063 |
| 1 | DCDC2 | 0.882 | 0.561 | 2.62E-299 | 0.935941 |
| 1 | PAGE4 | 0.191 | 0.029 | 2.93E-210 | 0.910853 |
| 1 | FN1 | 1 | 0.977 | 2.88E-210 | 0.862382 |
| 1 | VCAN | 1 | 0.975 | 1.19E-293 | 0.8189 |
| 1 | TPPP3 | 0.395 | 0.104 | 1.72E-280 | 0.795449 |
| 1 | ETFB | 0.976 | 0.908 | 1.22E-239 | 0.77577 |
| 1 | NR2F2 | 0.8 | 0.531 | 8.74E-206 | 0.770972 |
| 1 | GASK1B | 0.611 | 0.255 | 1.31E-257 | 0.762892 |
| 1 | MAP4K4 | 0.968 | 0.931 | 2.79E-150 | 0.727934 |
| 1 | SLIT3 | 0.58 | 0.289 | 7.91E-178 | 0.725999 |
| 1 | BEX5 | 0.4 | 0.261 | 4.92E-45 | 0.71483 |
| 1 | SERPINE2 | 0.736 | 0.456 | 2.02E-148 | 0.712743 |
| 1 | CCND2 | 0.98 | 0.92 | 9.80E-169 | 0.708745 |
| 1 | EPSTI1 | 0.479 | 0.191 | 2.31E-203 | 0.700716 |
| 1 | CYYR1 | 0.499 | 0.245 | 2.47E-159 | 0.69622 |
| 1 | HAS2 | 0.595 | 0.285 | 5.39E-174 | 0.686123 |
| 1 | FBLN2 | 0.514 | 0.171 | 2.09E-259 | 0.682068 |
| 1 | DPYSL3 | 0.789 | 0.654 | 1.35E-77 | 0.673731 |
| 1 | SLC2A3 | 0.776 | 0.51 | 3.61E-135 | 0.66176 |
| 2 | CXCL14 | 0.78 | 0.071 | 0 | 4.670497 |
| 2 | MIR205HG | 0.937 | 0.213 | 0 | 2.712285 |
| 2 | S100A3 | 0.576 | 0.073 | 0 | 2.316735 |
| 2 | FABP5 | 0.956 | 0.898 | 2.25E-156 | 2.216499 |
| 2 | NPY | 0.487 | 0.073 | 0 | 2.180049 |
| 2 | CCDC80 | 0.901 | 0.317 | 0 | 2.047503 |
| 2 | ARHGAP29 | 0.851 | 0.41 | 0 | 2.023437 |
| 2 | CRABP2 | 0.989 | 0.586 | 0 | 1.9889 |
| 2 | ANXA1 | 0.664 | 0.088 | 0 | 1.874636 |
| 2 | BMP4 | 0.739 | 0.128 | 0 | 1.841113 |
| 2 | ARL4C | 0.947 | 0.549 | 0 | 1.707661 |
| 2 | COL1A2 | 0.948 | 0.474 | 0 | 1.666447 |
| 2 | TFAP2B | 0.712 | 0.039 | 0 | 1.582863 |
| 2 | S100A9 | 0.101 | 0.008 | 5.94E-167 | 1.577778 |
| 2 | ADAMTS5 | 0.554 | 0.047 | 0 | 1.511961 |
| 2 | STC1 | 0.758 | 0.346 | 0 | 1.500886 |
| 2 | CDK6 | 0.87 | 0.47 | 0 | 1.498471 |
| 2 | MEIS2 | 0.983 | 0.756 | 0 | 1.474401 |
| 2 | WNT6 | 0.628 | 0.019 | 0 | 1.462856 |
| 2 | WLS | 0.966 | 0.854 | 0 | 1.45024 |
| 2 | SFN | 0.468 | 0.049 | 0 | 1.421455 |
| 2 | EFEMP1 | 0.69 | 0.132 | 0 | 1.415267 |
| 2 | SYNPO2 | 0.604 | 0.038 | 0 | 1.391273 |
| 2 | DLX5 | 0.619 | 0.024 | 0 | 1.385081 |
| 2 | COL5A2 | 0.734 | 0.116 | 0 | 1.38261 |
| 2 | BMP7 | 0.763 | 0.319 | 0 | 1.382441 |
| 2 | TPM1 | 0.999 | 0.99 | 0 | 1.381936 |
| 2 | MMP9 | 0.261 | 0.023 | 0 | 1.338517 |
| 2 | NET1 | 0.83 | 0.478 | 0 | 1.33828 |
| 2 | KRT7 | 0.427 | 0.176 | 2.67E-156 | 1.327358 |
| 3 | LCN15 | 0.861 | 0.147 | 0 | 4.572456 |
| 3 | AFP | 0.32 | 0.106 | 1.39E-149 | 4.554451 |
| 3 | GNRH2 | 0.482 | 0.039 | 0 | 4.308458 |
| 3 | APOA2 | 0.927 | 0.361 | 0 | 3.905241 |
| 3 | APOA1 | 0.931 | 0.494 | 0 | 3.862622 |
| 3 | CKB | 0.999 | 0.963 | 0 | 3.407218 |
| 3 | TTR | 0.781 | 0.412 | 4.07E-291 | 3.252864 |
| 3 | FGB | 0.251 | 0.025 | 0 | 3.120716 |
| 3 | IGFBP6 | 0.88 | 0.252 | 0 | 3.082922 |
| 3 | APOA4 | 0.101 | 0.013 | 2.46E-110 | 2.885224 |
| 3 | LGALS3 | 0.844 | 0.224 | 0 | 2.711253 |
| 3 | FABP1 | 0.266 | 0.134 | 3.53E-47 | 2.540421 |
| 3 | APOC3 | 0.133 | 0.056 | 6.18E-30 | 2.431893 |
| 3 | CST1 | 0.515 | 0.04 | 0 | 2.192997 |
| 3 | FN1 | 1 | 0.978 | 0 | 2.004816 |
| 3 | SERPINE2 | 0.893 | 0.448 | 0 | 1.919227 |
| 3 | FBLN2 | 0.806 | 0.15 | 0 | 1.917749 |
| 3 | RBP4 | 0.241 | 0.024 | 0 | 1.834013 |
| 3 | FGG | 0.185 | 0.009 | 0 | 1.829754 |
| 3 | SLC2A3 | 0.909 | 0.504 | 0 | 1.799002 |
| 3 | TF | 0.385 | 0.142 | 2.08E-153 | 1.796666 |
| 3 | NENF | 0.948 | 0.711 | 0 | 1.749585 |
| 3 | APOB | 0.355 | 0.155 | 1.15E-95 | 1.650781 |
| 3 | TFF1 | 0.216 | 0.046 | 1.35E-155 | 1.538058 |
| 3 | CST3 | 1 | 0.988 | 0 | 1.534802 |
| 3 | SAT1 | 0.991 | 0.896 | 0 | 1.531935 |
| 3 | SHH | 0.63 | 0.098 | 0 | 1.531338 |
| 3 | PRSS2 | 0.565 | 0.299 | 3.15E-112 | 1.530248 |
| 3 | KRT18 | 1 | 0.984 | 0 | 1.51588 |
| 3 | APOC1 | 0.726 | 0.496 | 7.88E-94 | 1.449754 |
| 4 | TOP2A | 0.997 | 0.43 | 0 | 2.383934 |
| 4 | CENPF | 0.992 | 0.506 | 0 | 2.246173 |
| 4 | UBE2C | 0.951 | 0.283 | 0 | 2.111425 |
| 4 | MKI67 | 0.963 | 0.286 | 0 | 2.084409 |
| 4 | NUSAP1 | 0.989 | 0.374 | 0 | 1.960066 |
| 4 | ASPM | 0.895 | 0.248 | 0 | 1.871586 |
| 4 | DLGAP5 | 0.952 | 0.263 | 0 | 1.85873 |
| 4 | TPX2 | 0.953 | 0.363 | 0 | 1.816758 |
| 4 | CCNB1 | 0.917 | 0.359 | 0 | 1.800165 |
| 4 | HMGB2 | 0.997 | 0.549 | 0 | 1.768797 |
| 4 | CDK1 | 0.943 | 0.31 | 0 | 1.662588 |
| 4 | CENPE | 0.832 | 0.259 | 0 | 1.661175 |
| 4 | PRC1 | 0.936 | 0.31 | 0 | 1.571097 |
| 4 | BIRC5 | 0.938 | 0.302 | 0 | 1.531907 |
| 4 | PTTG1 | 0.974 | 0.627 | 0 | 1.510881 |
| 4 | KIF11 | 0.903 | 0.275 | 0 | 1.488125 |
| 4 | CCNB2 | 0.84 | 0.253 | 0 | 1.46089 |
| 4 | SGO2 | 0.876 | 0.307 | 0 | 1.429415 |
| 4 | GTSE1 | 0.837 | 0.201 | 0 | 1.367315 |
| 4 | TACC3 | 0.848 | 0.245 | 0 | 1.349959 |
| 4 | KPNA2 | 0.924 | 0.592 | 2.02E-302 | 1.338933 |
| 4 | HMMR | 0.762 | 0.177 | 0 | 1.320319 |
| 4 | PBK | 0.881 | 0.25 | 0 | 1.287185 |
| 4 | H19 | 0.559 | 0.461 | 2.80E-35 | 1.286789 |
| 4 | ARL6IP1 | 0.995 | 0.95 | 3.14E-302 | 1.284183 |
| 4 | CALB1 | 0.435 | 0.162 | 7.41E-139 | 1.265265 |
| 4 | KIF20B | 0.911 | 0.384 | 0 | 1.264747 |
| 4 | CDC20 | 0.665 | 0.179 | 0 | 1.264178 |
| 4 | AURKA | 0.711 | 0.191 | 0 | 1.255954 |
| 4 | ECT2 | 0.881 | 0.374 | 0 | 1.254768 |
| 5 | GAL | 0.84 | 0.186 | 0 | 2.83142 |
| 5 | AREG | 0.789 | 0.221 | 0 | 2.642556 |
| 5 | SERPINA1 | 0.999 | 0.919 | 0 | 2.381188 |
| 5 | TIMP1 | 0.993 | 0.919 | 0 | 2.312675 |
| 5 | PMEPA1 | 0.931 | 0.45 | 0 | 1.963181 |
| 5 | ATF3 | 0.751 | 0.331 | 9.78E-284 | 1.916405 |
| 5 | KLF6 | 0.941 | 0.604 | 0 | 1.879021 |
| 5 | PDGFB | 0.816 | 0.303 | 0 | 1.842 |
| 5 | AKAP12 | 0.746 | 0.352 | 1.25E-226 | 1.817617 |
| 5 | MRC2 | 0.926 | 0.4 | 0 | 1.749678 |
| 5 | IGFBP5 | 0.916 | 0.696 | 7.12E-155 | 1.650647 |
| 5 | PON2 | 0.991 | 0.801 | 0 | 1.623518 |
| 5 | LYPD1 | 0.884 | 0.353 | 0 | 1.619127 |
| 5 | RHOB | 0.948 | 0.543 | 0 | 1.598089 |
| 5 | TGFB1 | 0.981 | 0.588 | 0 | 1.592307 |
| 5 | CXCL8 | 0.281 | 0.044 | 4.02E-237 | 1.582444 |
| 5 | BGN | 0.859 | 0.253 | 0 | 1.550139 |
| 5 | IGFBP7 | 0.936 | 0.482 | 0 | 1.54886 |
| 5 | SERPINA3 | 0.758 | 0.233 | 0 | 1.526408 |
| 5 | IER3 | 0.897 | 0.687 | 4.54E-207 | 1.501583 |
| 5 | NPW | 0.905 | 0.34 | 0 | 1.472192 |
| 5 | CTSZ | 0.836 | 0.431 | 0 | 1.469369 |
| 5 | DUSP1 | 0.707 | 0.259 | 0 | 1.463834 |
| 5 | CRLF1 | 0.768 | 0.258 | 0 | 1.460689 |
| 5 | TESC | 0.917 | 0.411 | 0 | 1.457609 |
| 5 | TCIM | 0.956 | 0.627 | 0 | 1.445446 |
| 5 | JUNB | 0.88 | 0.469 | 0 | 1.429882 |
| 5 | SPP1 | 0.89 | 0.55 | 4.82E-227 | 1.348997 |
| 5 | LEFTY1 | 0.595 | 0.152 | 0 | 1.295413 |
| 5 | RND1 | 0.632 | 0.192 | 0 | 1.280294 |
| 6 | CRABP1 | 0.524 | 0.098 | 0 | 3.686032 |
| 6 | PTN | 0.94 | 0.411 | 0 | 3.66019 |
| 6 | PTPRZ1 | 0.854 | 0.07 | 0 | 2.623853 |
| 6 | NR2F1 | 0.932 | 0.404 | 0 | 2.286106 |
| 6 | SFRP2 | 0.648 | 0.034 | 0 | 2.101507 |
| 6 | MAP1B | 0.987 | 0.702 | 0 | 1.913277 |
| 6 | SOX2 | 0.83 | 0.232 | 0 | 1.828432 |
| 6 | ZIC1 | 0.491 | 0.018 | 0 | 1.695366 |
| 6 | NEFM | 0.361 | 0.089 | 2.40E-187 | 1.659873 |
| 6 | FGFBP3 | 0.587 | 0.093 | 0 | 1.620184 |
| 6 | ZEB2 | 0.644 | 0.062 | 0 | 1.51782 |
| 6 | TMSB15A | 0.973 | 0.597 | 0 | 1.510977 |
| 6 | NEFL | 0.475 | 0.054 | 0 | 1.490287 |
| 6 | HES5 | 0.335 | 0.007 | 0 | 1.449624 |
| 6 | VIM | 1 | 0.948 | 0 | 1.439477 |
| 6 | PEG10 | 0.982 | 0.92 | 4.97E-142 | 1.432386 |
| 6 | PANTR1 | 0.521 | 0.009 | 0 | 1.397549 |
| 6 | HIST1H1D | 0.578 | 0.354 | 7.70E-88 | 1.392305 |
| 6 | METRN | 0.919 | 0.589 | 0 | 1.384726 |
| 6 | PRTG | 0.832 | 0.551 | 2.86E-202 | 1.379481 |
| 6 | POU3F2 | 0.539 | 0.044 | 0 | 1.339131 |
| 6 | TUBB2B | 0.995 | 0.891 | 0 | 1.303567 |
| 6 | FZD3 | 0.896 | 0.602 | 0 | 1.286246 |
| 6 | GAP43 | 0.295 | 0.044 | 2.76E-257 | 1.272973 |
| 6 | RMST | 0.43 | 0.077 | 0 | 1.222694 |
| 6 | HMGN2 | 0.999 | 0.973 | 3.75E-210 | 1.22184 |
| 6 | TUBA1A | 0.999 | 0.961 | 6.31E-262 | 1.203968 |
| 6 | HMGB2 | 0.89 | 0.562 | 4.41E-164 | 1.177889 |
| 6 | HIST2H2AC | 0.475 | 0.265 | 3.22E-79 | 1.165758 |
| 6 | TOP2A | 0.789 | 0.451 | 1.53E-108 | 1.155316 |
| 7 | CALB1 | 0.664 | 0.153 | 0 | 2.069207 |
| 7 | NKX6-2 | 0.634 | 0.171 | 1.97E-292 | 1.558897 |
| 7 | H19 | 0.669 | 0.456 | 3.05E-71 | 1.429815 |
| 7 | ONECUT1 | 0.995 | 0.6 | 1.52E-303 | 1.394989 |
| 7 | SLC4A4 | 0.925 | 0.472 | 1.24E-265 | 1.264102 |
| 7 | ANXA4 | 0.955 | 0.608 | 6.87E-256 | 1.223946 |
| 7 | ONECUT2 | 0.72 | 0.286 | 5.93E-199 | 1.172834 |
| 7 | CCND1 | 0.983 | 0.922 | 4.85E-138 | 1.141106 |
| 7 | CLU | 0.993 | 0.836 | 8.46E-200 | 1.117644 |
| 7 | AMBP | 0.984 | 0.701 | 1.96E-197 | 1.099869 |
| 7 | AC011139.1 | 0.422 | 0.081 | 9.63E-265 | 1.072505 |
| 7 | HABP2 | 0.729 | 0.272 | 6.57E-242 | 1.057018 |
| 7 | SPP1 | 0.638 | 0.572 | 1.21E-12 | 1.055086 |
| 7 | IGF2 | 0.989 | 0.744 | 1.99E-171 | 1.050554 |
| 7 | SFRP5 | 0.8 | 0.28 | 2.04E-267 | 1.015968 |
| 7 | FZD5 | 0.891 | 0.535 | 8.77E-175 | 0.998779 |
| 7 | CDH6 | 0.909 | 0.617 | 1.78E-140 | 0.946218 |
| 7 | RGS2 | 0.539 | 0.294 | 5.31E-68 | 0.936034 |
| 7 | GUCY1A2 | 0.79 | 0.489 | 4.54E-117 | 0.902924 |
| 7 | UPK1B | 0.475 | 0.128 | 1.50E-197 | 0.89846 |
| 7 | CD24 | 1 | 0.997 | 1.44E-238 | 0.88481 |
| 7 | SAMD5 | 0.722 | 0.318 | 2.21E-167 | 0.866456 |
| 7 | SPRY2 | 0.924 | 0.739 | 4.42E-142 | 0.848477 |
| 7 | TOX3 | 0.797 | 0.531 | 1.89E-118 | 0.823774 |
| 7 | LHFPL3-AS2 | 0.596 | 0.236 | 5.98E-147 | 0.799659 |
| 7 | LYZ | 0.459 | 0.159 | 1.86E-129 | 0.799395 |
| 7 | MECOM | 0.832 | 0.535 | 4.35E-129 | 0.787331 |
| 7 | NDFIP2 | 0.82 | 0.533 | 2.15E-129 | 0.776555 |
| 7 | ADGRG6 | 0.801 | 0.482 | 1.95E-115 | 0.75984 |
| 7 | LINC00261 | 0.837 | 0.591 | 2.77E-88 | 0.749694 |
| 8 | LTB | 0.929 | 0.139 | 0 | 3.843795 |
| 8 | IL32 | 0.972 | 0.3 | 0 | 3.512208 |
| 8 | AREG | 0.765 | 0.232 | 0 | 3.108937 |
| 8 | NPPB | 0.486 | 0.109 | 5.98E-268 | 3.099501 |
| 8 | PLAU | 0.718 | 0.103 | 0 | 2.370889 |
| 8 | TAGLN | 0.92 | 0.687 | 3.39E-220 | 2.222018 |
| 8 | TM4SF1 | 0.994 | 0.631 | 0 | 2.218327 |
| 8 | TACSTD2 | 0.964 | 0.413 | 0 | 2.12194 |
| 8 | CXCL8 | 0.382 | 0.042 | 0 | 2.050739 |
| 8 | S100A6 | 0.999 | 0.959 | 0 | 2.04341 |
| 8 | GDF15 | 0.716 | 0.372 | 5.69E-168 | 2.004313 |
| 8 | CCL2 | 0.375 | 0.06 | 1.15E-275 | 1.915174 |
| 8 | CGA | 0.57 | 0.128 | 0 | 1.831189 |
| 8 | AC022075.1 | 0.749 | 0.213 | 0 | 1.651015 |
| 8 | SDC4 | 0.966 | 0.564 | 0 | 1.639428 |
| 8 | PDGFB | 0.753 | 0.315 | 1.62E-223 | 1.631025 |
| 8 | CXCL12 | 0.79 | 0.24 | 0 | 1.619514 |
| 8 | RAB11FIP1 | 0.952 | 0.596 | 0 | 1.609861 |
| 8 | ACTC1 | 0.279 | 0.128 | 3.04E-39 | 1.584921 |
| 8 | ANKRD1 | 0.436 | 0.329 | 1.89E-15 | 1.579639 |
| 8 | ACTA1 | 0.206 | 0.029 | 1.66E-162 | 1.577883 |
| 8 | AKAP12 | 0.681 | 0.363 | 7.82E-115 | 1.570104 |
| 8 | IL11 | 0.57 | 0.113 | 0 | 1.555362 |
| 8 | CDH6 | 0.977 | 0.613 | 0 | 1.555028 |
| 8 | TNC | 0.756 | 0.302 | 1.68E-233 | 1.535558 |
| 8 | TMEM265 | 0.826 | 0.177 | 0 | 1.52326 |
| 8 | TESC | 0.936 | 0.418 | 0 | 1.478538 |
| 8 | MYL9 | 0.914 | 0.59 | 7.52E-219 | 1.45901 |
| 8 | CLDN6 | 0.995 | 0.819 | 0 | 1.434595 |
| 8 | NEAT1 | 0.996 | 0.974 | 1.45E-246 | 1.429404 |
| 9 | CHGA | 0.982 | 0.12 | 0 | 6.95249 |
| 9 | INS | 0.383 | 0.032 | 0 | 5.104942 |
| 9 | SST | 0.119 | 0.045 | 3.44E-20 | 4.835964 |
| 9 | CRYBA2 | 0.789 | 0.015 | 0 | 3.277838 |
| 9 | NEUROD1 | 0.934 | 0.011 | 0 | 3.185694 |
| 9 | C1QL1 | 0.955 | 0.043 | 0 | 3.177797 |
| 9 | STMN2 | 0.939 | 0.034 | 0 | 3.151428 |
| 9 | DDC | 0.91 | 0.038 | 0 | 3.138343 |
| 9 | INSM1 | 0.904 | 0.021 | 0 | 2.966475 |
| 9 | FEV | 0.908 | 0.014 | 0 | 2.954617 |
| 9 | SCGN | 0.976 | 0.193 | 0 | 2.922571 |
| 9 | ASCL1 | 0.68 | 0.017 | 0 | 2.921318 |
| 9 | SCG2 | 0.781 | 0.02 | 0 | 2.809784 |
| 9 | SCG3 | 0.926 | 0.063 | 0 | 2.619034 |
| 9 | PCSK1N | 0.994 | 0.702 | 0 | 2.562034 |
| 9 | C9orf16 | 0.991 | 0.866 | 0 | 2.452468 |
| 9 | MIR7-3HG | 0.881 | 0.013 | 0 | 2.431983 |
| 9 | CACNA2D1 | 0.883 | 0.06 | 0 | 2.422323 |
| 9 | CPE | 0.999 | 0.757 | 0 | 2.415722 |
| 9 | KCTD12 | 0.828 | 0.068 | 0 | 2.338746 |
| 9 | SEZ6L | 0.855 | 0.044 | 0 | 2.335642 |
| 9 | CXXC4 | 0.977 | 0.548 | 0 | 2.286293 |
| 9 | PCSK1 | 0.649 | 0.007 | 0 | 2.280192 |
| 9 | PPP1R1A | 0.877 | 0.181 | 0 | 2.20847 |
| 9 | RUNX1T1 | 0.831 | 0.065 | 0 | 2.15156 |
| 9 | ALDH1A1 | 0.904 | 0.239 | 0 | 2.097081 |
| 9 | GC | 0.409 | 0.017 | 0 | 2.043033 |
| 9 | SIM1 | 0.838 | 0.239 | 0 | 1.96672 |
| 9 | FABP7 | 0.544 | 0.07 | 0 | 1.9504 |
| 9 | PTPRN | 0.827 | 0.015 | 0 | 1.928723 |
| 10 | SOX2 | 0.916 | 0.24 | 0 | 2.044643 |
| 10 | IGFBP5 | 0.926 | 0.701 | 3.09E-133 | 1.613425 |
| 10 | PLEKHA5 | 0.908 | 0.623 | 2.24E-238 | 1.436514 |
| 10 | NPY | 0.376 | 0.106 | 1.10E-124 | 1.346548 |
| 10 | ANGPT2 | 0.743 | 0.358 | 1.18E-180 | 1.325598 |
| 10 | CLDN18 | 0.319 | 0.156 | 2.72E-38 | 1.256242 |
| 10 | SULF1 | 0.358 | 0.107 | 5.45E-120 | 1.223218 |
| 10 | PTPN13 | 0.669 | 0.253 | 1.41E-216 | 1.192291 |
| 10 | TXNIP | 0.973 | 0.773 | 1.21E-216 | 1.16848 |
| 10 | HOTAIRM1 | 0.696 | 0.308 | 9.19E-174 | 1.149465 |
| 10 | SOX21 | 0.576 | 0.076 | 0 | 1.110427 |
| 10 | TNNT1 | 0.81 | 0.403 | 3.62E-182 | 1.108047 |
| 10 | SPINK5 | 0.301 | 0.036 | 1.59E-269 | 1.107743 |
| 10 | PCDH19 | 0.435 | 0.057 | 0 | 1.072445 |
| 10 | CADM1 | 0.95 | 0.902 | 4.58E-91 | 1.040017 |
| 10 | DMD | 0.566 | 0.185 | 1.66E-195 | 1.034482 |
| 10 | LINC02381 | 0.925 | 0.65 | 1.84E-181 | 1.02006 |
| 10 | CEACAM6 | 0.373 | 0.05 | 2.04E-302 | 0.97752 |
| 10 | FRZB | 0.326 | 0.099 | 6.00E-98 | 0.972747 |
| 10 | CD9 | 0.711 | 0.245 | 1.43E-220 | 0.943281 |
| 10 | TMSB4X | 1 | 1 | 8.94E-138 | 0.93442 |
| 10 | KLF5 | 0.702 | 0.427 | 1.51E-97 | 0.928805 |
| 10 | AGR3 | 0.387 | 0.055 | 5.72E-298 | 0.926341 |
| 10 | C19orf33 | 0.55 | 0.226 | 2.89E-120 | 0.89594 |
| 10 | NUDT4 | 0.814 | 0.59 | 8.33E-83 | 0.894451 |
| 10 | MIR205HG | 0.671 | 0.275 | 1.16E-133 | 0.871283 |
| 10 | SCNN1A | 0.639 | 0.222 | 2.51E-201 | 0.861507 |
| 10 | IGDCC3 | 0.888 | 0.646 | 2.39E-118 | 0.855897 |
| 10 | PLA2G2A | 0.151 | 0.125 | 1 | 0.788237 |
| 10 | NR2F2 | 0.771 | 0.554 | 8.88E-67 | 0.782999 |
| 11 | COL3A1 | 0.883 | 0.097 | 0 | 4.132443 |
| 11 | LGALS1 | 0.914 | 0.26 | 0 | 3.818895 |
| 11 | COL1A1 | 0.955 | 0.479 | 2.29E-240 | 3.425046 |
| 11 | LUM | 0.777 | 0.035 | 0 | 3.406115 |
| 11 | ACTA2 | 0.604 | 0.231 | 3.34E-105 | 3.214171 |
| 11 | ACTC1 | 0.392 | 0.13 | 4.88E-63 | 2.976214 |
| 11 | COL1A2 | 0.959 | 0.516 | 4.90E-225 | 2.908414 |
| 11 | COL6A3 | 0.682 | 0.039 | 0 | 2.781894 |
| 11 | TAGLN | 0.968 | 0.694 | 1.90E-164 | 2.628687 |
| 11 | ITGA1 | 0.75 | 0.156 | 1.16E-298 | 2.525444 |
| 11 | COL4A1 | 0.806 | 0.385 | 1.42E-136 | 2.508119 |
| 11 | SPARC | 0.964 | 0.54 | 2.82E-197 | 2.484634 |
| 11 | CCN2 | 0.775 | 0.399 | 7.88E-97 | 2.456752 |
| 11 | TPM2 | 0.991 | 0.761 | 2.00E-215 | 2.373501 |
| 11 | ESM1 | 0.131 | 0.003 | 1.75E-224 | 2.312091 |
| 11 | DCN | 0.493 | 0.015 | 0 | 2.238922 |
| 11 | TPM1 | 0.998 | 0.991 | 3.50E-155 | 2.235557 |
| 11 | GNG11 | 0.831 | 0.084 | 0 | 2.23294 |
| 11 | ANXA1 | 0.727 | 0.138 | 7.43E-286 | 2.209857 |
| 11 | CDH11 | 0.829 | 0.102 | 0 | 2.199465 |
| 11 | TIMP3 | 0.752 | 0.179 | 5.19E-236 | 2.17102 |
| 11 | IGFBP7 | 0.851 | 0.507 | 1.22E-120 | 2.110698 |
| 11 | CALD1 | 1 | 0.966 | 3.88E-182 | 2.105211 |
| 11 | PAPPA | 0.403 | 0.147 | 1.50E-56 | 2.079364 |
| 11 | MT2A | 0.473 | 0.046 | 0 | 2.062394 |
| 11 | MYL9 | 0.939 | 0.6 | 1.24E-167 | 2.060777 |
| 11 | VIM | 1 | 0.951 | 8.04E-212 | 2.027305 |
| 11 | COL5A2 | 0.883 | 0.167 | 0 | 2.017326 |
| 11 | COL5A1 | 0.707 | 0.227 | 6.52E-154 | 1.950573 |
| 11 | CCDC80 | 0.876 | 0.371 | 4.50E-162 | 1.928981 |

**References**

1. Pombo-Suarez, M.; Calaza, M.; Gomez-Reino, J. J.; Gonzalez, A., Reference genes for normalization of gene expression studies in human osteoarthritic articular cartilage. *BMC molecular biology* **2008,** *9* (1), 1-7.

2. Hildebrand, L.; Rossbach, B.; Kühnen, P.; Gossen, M.; Kurtz, A.; Reinke, P.; Seemann, P.; Stachelscheid, H., Generation of integration free induced pluripotent stem cells from fibrodysplasia ossificans progressiva (FOP) patients from urine samples. *Stem Cell Research* **2016,** *16* (1), 54-58.

3. Alexander, N. R.; Tran, N. L.; Rekapally, H.; Summers, C. E.; Glackin, C.; Heimark, R. L., N-cadherin gene expression in prostate carcinoma is modulated by integrin-dependent nuclear translocation of Twist1. *Cancer research* **2006,** *66* (7), 3365-3369.

4. Messai, Y.; Noman, M. Z.; Derouiche, A.; Kourda, N.; Akalay, I.; Hasmim, M.; Stasik, I.; Ben Jilani, S.; Chebil, M.; Caignard, A., Cytokeratin 18 expression pattern correlates with renal cell carcinoma progression: relationship with Snail. *International journal of oncology* **2010,** *36* (5), 1145-1154.

5. Lou, C.; Zhang, F.; Yang, M.; Zhao, J.; Zeng, W.; Fang, X.; Zhang, Y.; Zhang, C.; Liang, W., Naringenin decreases invasiveness and metastasis by inhibiting TGF-β-induced epithelial to mesenchymal transition in pancreatic cancer cells. *PloS one* **2012,** *7* (12), e50956.

6. Velazco-Cruz, L.; Song, J.; Maxwell, K. G.; Goedegebuure, M. M.; Augsornworawat, P.; Hogrebe, N. J.; Millman, J. R., Acquisition of dynamic function in human stem cell-derived β cells. *Stem cell reports* **2019,** *12* (2), 351-365.

7. Taylor, B. L.; Liu, F.-F.; Sander, M., Nkx6. 1 is essential for maintaining the functional state of pancreatic beta cells. *Cell reports* **2013,** *4* (6), 1262-1275.

8. Liu, Q.; Zhang, X.; Dai, L.; Hu, X.; Zhu, J.; Li, L.; Zhou, C.; Ao, Y., Long noncoding RNA related to cartilage injury promotes chondrocyte extracellular matrix degradation in osteoarthritis. *Arthritis & rheumatology* **2014,** *66* (4), 969-978.

9. McGrath, P. S.; Watson, C. L.; Ingram, C.; Helmrath, M. A.; Wells, J. M., The basic helix-loop-helix transcription factor NEUROG3 is required for development of the human endocrine pancreas. *Diabetes* **2015,** *64* (7), 2497-2505.
